# Effects of gallium and clove oil embedded in porous phosphate coacervate glass fibres on wound healing

**DOI:** 10.1039/d6ma00177g

**Published:** 2026-06-11

**Authors:** Zarrin Moghaddam, Rahul Sanwlani, Pejman Ghaffari, Irem Unalan, James R. G. Adams, Roberto di Pasquale, Charlotte A. Berry, Hongjuan Zhao, Monica Felipe-Sotelo, Aldo R. Boccaccini, Armin Shavandi, Patrizia Camelliti, Daniela Carta

**Affiliations:** a School of Chemistry and Chemical Engineering, Faculty of Engineering and Physical Sciences, University of Surrey Guildford GU2 7XH UK d.carta@surrey.ac.uk; b School of Biosciences, Faculty of Health and Medical Sciences, University of Surrey Guildford GU2 7XH UK; c Université libre de Bruxelles (ULB), École Polytechnique de Bruxelles 3BIO-BioMatter, Avenue F.D. Roosevelt, 50 - CP 165/61 1050 Brussels Belgium; d Institute of Biomaterials, Department of Materials Science and Engineering, Friedrich-Alexander-University Erlangen-Nuremberg Caustraße 6 91058 Erlangen Germany; e School of Veterinary Medicine, Faculty of Health and Medical Sciences, University of Surrey Guildford GU2 7XH UK

## Abstract

This study investigates Ga-loaded porous phosphate glass fibres (PGFs) coated with clove oil (clv) (1.5 and 3 w/v%) as multifunctional materials capable of antioxidant, antibacterial, and wound-healing properties. PGFs were prepared *via* coacervation combined with supramolecular templating and electrospinning (ES). Porosity was induced *via* the removal of the surfactant cetyltrimethylammonium bromide (CTAB) used as a soft template. Scanning Electron Microscopy (SEM) revealed that the majority of porous PGFs have diameters ranging from ∼0.5 to 4 µm. PGFs around 1–4 µm have highly porous walls (pores ∼300 nm in diameter), whereas the smaller ones are hollow, with non-porous walls and perfectly round channels. Results have identified PGFs loaded with 1 mol% Ga_2_O_3_ and 3 w/v% clv as ideal compositions, exhibiting the strongest radical scavenging (up to ∼70% DPPH, ∼53% ABTS), the highest inhibition of hydroxyl radicals (OH˙) (∼34% reduction) and the highest phenolic content (∼18 mg GAE/g). Reactive oxygen species (ROS) assays demonstrated that PGFs’ dissolution products show a protective effect under oxidative stress by significantly reducing intracellular ROS levels while maintaining high keratinocyte (HaCaT) viability over 72 h. Similarly, in fibroblast-like (MC3T3-L1-E1), the same PGFs’ dissolution products enhanced H_2_O_2_ scavenging activity and improved cytocompatibility. Time-dependent MTS assays in HaCaTs demonstrated sustained cell viability over 72 h across two tested concentrations (5% and 10% v/v), with stable metabolic activity and no evidence of significant cytotoxicity. Antibacterial activity against *Escherichia coli* (*E. coli*) was confirmed by optical density (OD) measurements and colony-forming unit (CFU) analysis, which showed a significant reduction in viable bacteria for PGFs containing 1 mol% Ga_2_O_3_ coated with 3 w/v%. This antibacterial effect is hypothesised to result from the release of Ga ions, while clv contributes mainly to the antioxidant activity. In contrast, PGFs’ dissolution products, regardless of Ga and clv loading, did not exhibit a measurable antibacterial effect against *Staphylococcus aureus* (*S. aureus*). *In vitro* wound healing scratch assays showed that dissolution products from Ga- and clv-containing PGFs enhance cell migration, accelerating wound closure in both keratinocytes and fibroblasts. This work demonstrates the successful synthesis of porous PGFs by combining soft templating with the coacervation method and the enhanced beneficial properties of PGFs coated with a natural antioxidant, proving their potential for advanced wound-healing applications. Further *in vivo* studies will be required to confirm the *in vitro* potential.

## Abstract 2

This study investigates Ga-loaded porous phosphate glass fibres coated with clove oil as multifunctional materials capable of antioxidant, antibacterial, and wound-healing properties.

## Graphical abstract

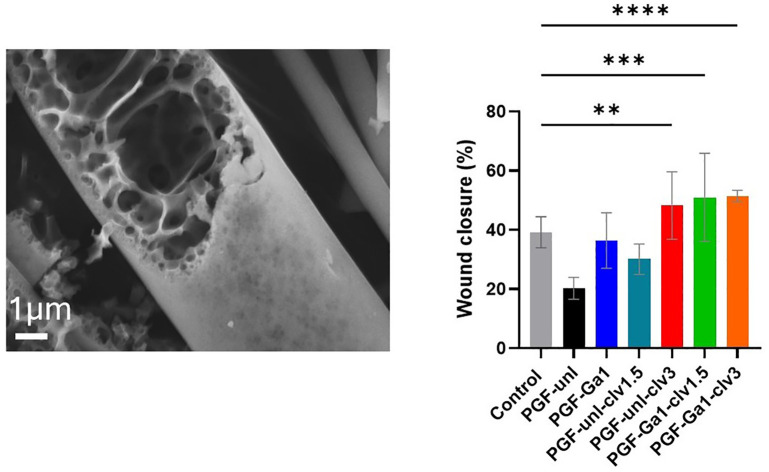

## 1. Introduction

Recent advances in wound-healing strategies have increasingly focused on multifunctional biomaterials capable of actively modulating the wound microenvironment.

There is a clear unmet urgent clinical need for affordable, bioresorbable wound repair materials capable of both stimulate healing and delivering sustained antibacterial activity preventing and treating infections. Inorganic biomaterials such as silicate-based glasses, borate-based glasses and phosphate-based glasses (PGs) have gained interest in recent years for their applications in wound healing and drug delivery due to their ability to release biologically active ions that stimulate cellular responses.^1^

PGs have gained significant interest due to their enhanced solubility compared to silicate-based glasses and lower toxicity compared to the borate ones,^2,3^ enabling their use in drug delivery and tissue engineering. Their ability to degrade in physiological environments (bioresorbability) makes them particularly attractive for wound healing applications.^4^

Currently, products that provide a scaffold for wound repair (matrices) include *skin autograft* (transfer of healthy patient's own skin), *skin allograft* (donor's skin*) or xenograft* (animal tissue). These matrices have severe limitations: pain, prolonged surgery, uncontrollable bioresorption rate, rejection, limited availability, variable immune responses and transmission of diseases.^5^ Biological acellular/de-cellularised matrices are often used to minimise inflammatory/immunogenic responses and improve shelf-life/storage conditions. However, the de-cellularisation process is expensive, delivers variable quality and frequently leads to rapid degradation. Fully synthetic products overcome these limitations, avoiding risk of disease transmission, and offering control of the composition/morphology. However, synthetic matrices, often based on polymers (*e.g* polyglycolic/polylactic acid, polycaprolactone) can cause toxicity and inflammation due to accumulation of crystalline/acidic degradation products.^6^ In addition, natural polymers (*e.g.* gelatin, collagen, chitosan, hyaluronic acid), synthetic polymers and hydrogels (hydrophilic polymers) can often have low biocompatibility (*e.g.* due to residual solvents), unpredictable solubility, poor wettability, poor durability and lack of cell-binding sites. For example, ionic hydrogels containing divalent cations (*e.g.* Ca^2+^) can undergo ion exchange with monovalent cations in the body, resulting in collapse of the hydrogel and degradation by enzymes, limiting their use as drug delivery or tissue scaffolds.^7^ For these reasons, polymers and hydrogels are frequently functionalised and/or combined with biological materials in composite matrices.^8^ Synergistic platforms, such as hydrogel-exosome composites have been designed to overcome the limitations of natural hydrogels.^9^ However, there are challenges associated with their development and applications such as standardisation of the exosome extraction, purification processes and optimisation of their long-term stability.^9^

One key parameter in selecting materials for wound-healing applications is the water vapour transmission rate (WVTR), which plays a critical role in maintaining the optimal moisture balance within the wound environment. Polymeric membrane dressings (polyurethane) were reported to maintain an optimal moisture content of ∼2028.3  ±  237.8 g m^−2^ d^−1^.^10^ Hydrocolloids and hydrogels were reported to have WVTR in the range 6500–76 and 9000–50 g m^−2^ d^−1^, respectively.^11^

Data on glasses are very limited; a WVTR of a silicate-based glass was reported to be 6700 g m^−2^ d^−1^.^12^ To the knowledge of the authors, there is no available WVTR data on PGs.

PGs prepared *via* coacervation are a promising alternative to fully polymeric bioinks as they have intrinsic biocompatibility and bioactivity, better stability and durability. Moreover, active species, such as antibacterial ions can be added directly into the coacervate gel achieving uniform distribution and therefore improving therapeutic performance.

A clear advantage of PGs over polymers, in particular hydrogels, is the incorporation of porosity. Porous PGs can be manufactured relatively easily in comparison to porous polymers, further enhancing PGs functionalities by increasing surface area, improving ion exchange, promoting controlled release and facilitating absorption of therapeutic molecules/ions.^13^ On the contrary, synthesis of porous hydrogels presents several challenges related to complex manufacturing, control of shape and distribution of pores, and stability.^14^

PGs can also be easily manufactured in fibrous morphology *via* ES of water-based phosphate-based coacervate precursors. Crucially, unlike ES of most of polymers (*e.g.* chitosan, cellulose or gelatin), ES of phosphate-based precursors does not require toxic or acidic solvents.^15^

PGFs are particularly suitable for wound healing applications, as they mimic the structure of the natural extracellular matrix.^14^ PGFs have an intrinsic intra-fibre porosity due to the void spaces in between the fibres. This type of porosity (usually macroporosity - pore diameter >50 nm) promotes cell attachment, tissue growth, and vascularization. However, additional intra-fibres porosity (microporosity - pore diameter <2 nm and mesoporosity - pores diameter between 2 and 50 nm) is desirable as pores within the fibres can act as hosts of therapeutic molecules/ions and enhance their absorption/release.^2^ Release of therapeutic species to surrounding body fluids stimulates cellular responses and supports tissue regeneration.^1,3^

Porous glasses are usually produced *via* the sol–gel (SG) route as the in-solution chemistry of hydrolysis and polycondensation combined with supramolecular templating lends itself naturally to forming networks with tuneable pore architectures.^5^ Templating is a consolidated method for fabricating porous silicate-based glasses enabling the development of tailored morphologies and geometries by utilizing soft templates, such as surfactants, during the synthesis.^15^ Porous SG-derived silicate glasses have been reported to enhance bone regeneration by facilitating vascularization and transport of nutrients.^6,7^

Manufacturing porous PGs is more challenging than manufacturing porous silicate-based glasses due to the inherent weakness of the phosphate network, which is prone to collapse and crystallize at the temperatures usually required to generate porosity.^11^ Nevertheless, porous PGs prepared *via* SG in the systems P_2_O_5_–CaO–Na_2_O,^12^ P_2_O_5_–CaO–Na_2_O–ZnO,^16^ P_2_O_5_–CaO–Na_2_O–Cu_*x*_O_*y*_^17^ and P_2_O_5_–CaO–Na_2_O–SrO^18^ using the non-ionic block copolymer surfactant Pluronic 123 (P123) have been reported. More recently, P_2_O_5_–CaO–Na_2_O–CuO and P_2_O_5_–CaO–Na_2_O–Ga_2_O_3_ manufactured *via* SG using a different block copolymer surfactant, Pluronic F108, have been investigated.^19^ However, the SG technique has some disadvantages such as the use of organic precursors often toxic and long gelation times. On the other hand, the most common melt-quenching (MQ) process for PGs production is not ideal for manufacturing porous PGs due to the high temperatures involved.^4^

In this work, porous PGFs were fabricated by using a sustainable, cost-effective in-solution technique (coacervation) followed by ES. This technique consists of the gradual addition of a solution of M^2+^ ions to an aqueous solution of sodium polyphosphate under continuous stirring;^4^ this causes the formation of two phases, a supernatant aqueous phase and a polyphosphate coacervate gel-like bottom phase. The bottom gel phase can be electrospun to obtain PGFs or dried in a desiccator to obtain powders.^4,13^ The coacervate method is an effective alternative approach to SG and MQ, offering control over composition, versatility and green and mild synthesis conditions (room temperature, water-based). A polyphosphate gel embedded with calcium, sodium, gallium ions and the templating agent CTAB was injected into an electrospinner to generate porous PGFs. Inter-fibres porosity was induced by subsequent CTAB removal *via* calcination. CTAB, a widely used cationic surfactant, has proven to be a highly effective soft template for generating porous structures.^16^ The surfactant molecules self-assemble into micellar structures, which act as templates for the glass network to form, leading to well-defined porous architectures upon removal of the template. This study introduces a novel and sustainable coacervation-assisted ES approach combined with CTAB templating to fabricate porous PGFs, overcoming key limitations associated with conventional SG and MQ methods.

In addition, ES of surfactant-loaded gel precursors opens new opportunities for inducing aligned porosity, as the surfactants can align themselves with the electrical field during ES. Given the expected enhanced adsorption capacity, porous PGFs were embedded with the natural component clv to further improve their therapeutic effectiveness. Clv, which mainly contains the active ingredient eugenol, is well known for its antioxidant and antibacterial properties, and has been incorporated into various biomaterials to control infection while preserving cytocompatibility.^20^ Previous investigation of calcium silicate-based mesoporous glass nanoparticles impregnated with clv have shown high encapsulation efficiency, sustained release, enhanced inhibition of *E. coli* and *S. aureus*, and enhanced MG-63 osteoblast-like cells viability.^21^ Recently, porous clv-loaded PGFs in the system P_2_O_5_–CaO–Na_2_O–Ga_2_O_3_ (0.2, 0.5, and 1 mol% Ga_2_O_3_), synthesised using P123 as a surfactant, were also reported to exhibit antioxidant activity, with the antioxidant response increasing with Ga loading.^4^

However, despite these promising findings, the biological performance of these materials in wound-healing-relevant models remains largely unexplored. In particular, the ability of Ga/clv-loaded PGFs to modulate oxidative stress in skin cells, maintain long-term cytocompatibility, support cell migration and wound closure, and provide antibacterial activity has not been systematically investigated.

Therefore, the present work focuses on the biological validation of porous Ga/clv-loaded PGFs through extended cytocompatibility studies in keratinocytes over 72 h, intracellular ROS assays under oxidative stress conditions, antibacterial evaluation against both Gram-negative and Gram-positive bacteria, and wound closure assessment in both fibroblast-like and keratinocyte cell models. Moreover, a different porogen (CTAB), was used in this work. By combining these complementary morphological and biological investigations, this study provides a comprehensive evaluation of the wound-healing-related potential of porous Ga/clv-loaded PGFs.

Given the suitability of fibrous structures for wound healing applications, the antioxidant, antibacterial, and oxidative stress-modulating properties of the materials were investigated in two skin-related cell models, keratinocytes (HaCaTs) and fibroblasts-like (MC3T3-L1-E1). Ga^3+^ and clv have been reported in literature to have a positive effect on cell migration and wound closure. Dissolution products from silicate-based bioactive glasses containing ∼1 mol% Ga_2_O_3_ have been shown to promote Saos-2 cell migration *via* the *in vitro* scratch test, with the simulated wound largely closing within 24 h and reaching complete closure by ∼3 days.^22^ Clove oil emulsions have also been reported to accelerate *in vitro* wound closure by increasing fibroblast migration, suggesting a direct pro-healing effect in addition to their antibacterial activity.^23^

The proposed method enables improved control over intra-fibre porosity, enhancing the loading and release of clv. Furthermore, the combined release of Ga^3+^ and clv demonstrates an additive effect in accelerating MC3T3–L1–E1 and HaCaTs migration, highlighting the potential of this system as a multifunctional platform for wound healing applications.

The novelty of the present work lies in the comprehensive biological evaluation of porous Ga/clove-loaded PGFs produced using CTAB as a porogen, including long-term cytocompatibility, oxidative stress modulation, antibacterial activity and wound-healing-related behaviour.

*In vivo* studies will be needed to confirm this potential in a 3D model.

## 2. Experimental

### 2.1. Synthesis

To synthesise porous PGFs, 0.6 g of CTAB (Sigma-Aldrich, ≥96%) was dissolved in 95 mL water and 5 mL ethanol (C_2_H_5_OH, Sigma-Aldrich, ≥99.9%). A low CTAB concentration (around 8.2 mM) was chosen to minimize carbon contamination typically associated with higher surfactant levels.^24^ 47.2 g of calcium nitrate (Ca (NO_3_)_2_·4H_2_O, Fisher, >99%) were then added to the 100 mL CTAB solution. 20 mL of this mixture were then added to 20 mL of a 0.16 M aqueous solution of sodium polyphosphate (Na(PO_3_)_*n*_, *n* ≃ 25, Thermo Scientific) using a syringe pump at a rate of 20 mL h^−1^. The mixture was stirred for 1 h. To prepare the Ga-loaded PGFs, 0.24, 0.6 and 1.2 mL of 2 M gallium(iii) nitrate hydrate (Ga(NO_3_)_3_·H_2_O, Alfa Aesar) were added to the mixture to synthesise PGFs with 0.2, 0.5 and 1 mol% Ga_2_O_3_ content, respectively.

Once isolated, the coacervate gels were injected into a nozzle and electrospun. ES was performed at room temperature. The distance between the nozzle and the collector was set at 15 cm, the flow rate was set at 2 mL h^−1^ and a voltage of 15–18 kV was applied between the nozzle and a metal screen collector, where PGFs were deposited. This range was selected based on previous studies on the fabrication of PGFs using ES.^25,26^ PGFs were then calcined at 350 °C to remove CTAB and produce porosity.

To coat the PGF-Ga with clv (Sigma Aldrich), 100 mg of calcined PGFs were immersed in a mixture of ethanol and clv (41 and 81 µL of clv in ∼3 mL of ethanol, corresponding to nominal clv concentrations of ∼1.5 and ∼3 w/v%, respectively) for 24 h. The mixture was centrifuged at 4000 rpm at 5 °C for 15 minutes (min). The supernatant was then removed and the remaining PGFs were left in fume hood until the ethanol evaporated.

Samples will be hereafter named as PGF-unl (PGFs unloaded) and PGF-Ga*X* for PGFs loaded with Ga, where *X* is the nominal Ga_2_O_3_ mol% content (*X* = 0.2, 0.5 and 1). PGFs coated with clv will be named as PGF-unl-clv for the Ga free samples and PGF-Ga*X*-clv for the Ga loaded ones, respectively. All PGFs were then calcined at 350 °C with a heating rate of 1 °C min^−1^ and held at this temperature for 30 min.

### 2.2. Characterisation

SEM images were obtained on a JSM-7100F (Jeol, Japan) at an accelerating voltage of 15 kV. PGFs were carbon coated to reduce charging and mounted onto an aluminium stub using carbon conductive tape. The Fiji software was used to analyse the pore size and diameter of PGFs.^27^

EDX was performed using a WDS MagnaRay spectrometer (UK). Five points on the PGFs’ surfaces were selected to analyse the elemental composition, and values are reported as mean ± standard deviation (SD).

XRD was performed using a PANalytical X’Pert spectrometer (Malvern Panalytical, The Netherlands) using a flat plate geometry and Ni filtered Cu-Kα radiation. Data was collected using a PIXcel-1D detector with a step size of 0.0525° and a time per step of 12 seconds over an angular range of 2*θ* = 10–90°.

ATR-FT-IR spectra were collected using a PerkinElmer 2000 FT-IR spectrometer (PerkinElmer Inc., USA) over the range 1500–500 cm^−1^, with 16 scans per spectrum.

### 2.3. Dissolution study

The dissolution behaviour of PGFs was assessed by analysing the ions released after immersion in deionized (DI) water at different time intervals. Release tests were performed by immersing 10 mg of each PGF in 10 mL of deionised (DI) water, followed by incubation in a shaker at 37 °C and 220 rpm for 3, 24, 48, and 72 h. After each time point, the suspensions were centrifuged at 4500 rpm for 5 min, and the supernatants were filtered using a 0.45 µm Millex™-GP filter unit. Each experiment was performed in triplicate. The concentrations of phosphorus (P), calcium (Ca), sodium (Na), and gallium (Ga) were determined using microwave plasma atomic emission spectroscopy (MP-AES, 4210 Agilent, USA). Samples for MP-AES analysis were prepared by diluting 200 µL of the filtered dissolution products to a final volume of 10 mL in of 2% v/v nitric acid (HNO_3_, trace metal analysis grade, Fisher Scientific). The emission signals of the analytes were measured at 213, 393, 588 and 417 nm for P, Ca, Na and Ga, respectively. The emission of each of the analytes was normalised by the signal of the Be (measured at 234 nm) used as internal standard and added to all standards and samples *via* a T-connection prior nebulisation (5 µg mL^−1^, prepared from a 1000 µg mL^−1^ stock purchased from PalmaCAL). Standards of the four analytes at concentrations of 0.5, 1, 2, 5, 10, 25 and 50 µg mL^−1^ were freshly prepared before calibration by dilution with 2% v/v HNO_3_ from individual commercial 1000 µg mL^−1^ stock solutions (PalmaCAL). The instrumental limits of detection (LOD, based on the 3 × SD_blank_ criterion) were 0.2, 0.1, 0.1 and 0.4 µg mL^−1^ for P, Ca, Na and Ga respectively.

Dissolution studies have been performed in DI water to minimise interferences in solution. In addition, dissolution studies have also been performed in Simulated Body Fluid (SBF), as a more physiologically representative dissolution medium. The SBF solution was prepared according to the method reported by Kokubo *et al.*^28^ Ion release after 3, 24 and 48 h was measured, and the solutions were acidified, quantified, and corrected using the same protocols as described above for samples dissolved in DI water.

### 2.4. Release of clv from PGFs

The release profile of clv from PGFs was monitored using UV-visible spectroscopy (Specord 40, Analytik Jena AG, Germany). PGFs coated with clv were immersed in 10 mL of Dulbecco's phosphate-buffered saline (PBS, Gibco™) and incubated at 37 °C in an orbital shaker set at 90 rpm. At different time points (1, 3, 6, 12, 24, 48, 72, and 168 h), 0.5 mL of the supernatant were collected for analysis, and an equal volume of fresh PBS was added to maintain a constant solution volume. The absorbance of the collected samples was measured at 280 nm to determine eugenol (C_10_H_12_O_2_, Sigma-Aldrich) concentration, with a calibration curve generated using standard eugenol solutions ranging from 0.6 to 9.7 µg mL^−1^. Measurements were conducted in triplicate.

### 2.5. Antibacterial activity

The antibacterial activity of PGFs was studied by analysing the effect of their dissolution products after 24 h of immersion in DI water on the growth of *S. aureus* and *E. coli* (*K*12),^29^ two strains of bacteria commonly found in wounds. They were grown aerobically in Tryptone Soya Broth (TSB, Oxoid) at 37 °C on an orbital shaker (250 rpm) until the cultures reached the mid-exponential phase. The cells were then diluted to an optical density of 0.05 at 600 nm (OD_600_) in a 96-well plate with TSB containing the 24 h dissolution products of PGFs at the ratio of 1 : 10 (10 µL of PGFs in 90 µL of bacterial culture) or sterile water blank (10 µL of double-distilled H_2_O in 90 µL of bacterial culture) in triplicates and incubated again at 37 °C.

For the quantification of the OD_600_ plates were incubated for 24 h before each well was measured using a CLARIOstar plate reader (BMG LABTECH, Germany). The OD_600_ measures the turbidity of a given solution, which is positively correlated with the concentration of bacteria present in the broth after each treatment. The values obtained were corrected with a blank containing only TSB.

For the determination of bacterial viability, plates were incubated for 1 h and bacterial counts were determined using the Miles and Misra technique.^30^ Briefly, bacterial cultures were serial diluted 10-fold in sterile PBS and 20 µL of each dilution was dropped in triplicate onto nutrient agar before incubation for 16 h at 37 °C, aerobically. Colonies were then counted and colony-forming units (CFU) per mL were calculated.

### 2.6. Cell viability of HaCaTs in contact with PGFs

Cell viability was assessed using the MTS assay in HaCaTs following exposure to PGFs' dissolution products. Cells were seeded in 96-well plates at a density of 5 × 10^3^ cells per well in Dulbecco Modified Eagle Medium (DMEM) supplemented with l-glutamine, penicillin/streptomycin (Pen/Strep), 10% fetal bovine serum (FBS), and 1 mM CaCl_2_, and incubated at 37 °C in a humidified atmosphere containing 5% CO_2_ for 24 h. The culture medium was then replaced with fresh medium containing PGFs' dissolution products at two concentrations (5% and 10% v/v), prepared by adding 20 µL or 40 µL of dissolution products to 380 µL or 360 µL of medium, respectively. For PGF-clv1.5 samples, HaCaTs viability was assessed after 24 h of exposure. For PGF-clv3, cell viability was evaluated over extended timepoints (0, 24, 48, and 72 h) to assess potential time-dependent effects. At each timepoint, 20 µL of MTS reagent was added per well and incubated for 1 h at 37 °C.

Cell viability (%) was calculated using the following equation:1
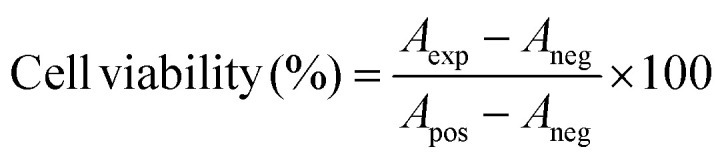
where *A*_exp_ is the absorbance of treated cells, *A*_neg_ is the absorbance of the cell-free medium (negative control), and *A*_pos_ is the absorbance of untreated cells (positive control), all measured at 490 nm using a microplate reader (SpectraMax iD3, US). For time-dependent assays, the application of a background correction (490–630 nm) consistently improved measurement accuracy. Experiments were performed in triplicate across three independent biological replicates (*N* = 3) and three technical triplicates (*n* = 3).

### 2.7. DPPH, ABTS and TMB assay for PGFs coated with clv

The free radical scavenging activity of PGFs was studied using the DPPH assay (1,1-diphenyl-picryl-hydrazine, C_18_H_13_N_5_O_6_, Sigma-Aldrich) performed according to Unalan *et al.*^31^ and the ABTS assay (2,2′-azino-bis(3-ethylbenzothiazoline-6-sulfonic acid)) (ABTS, >98%, TCI), following the procedure reported by Ghaffari *et al.*^32^

The DPPH assay was performed in a dark room due to the DPPH radical being light sensitive. 1 mg of coated PGFs was immersed in 1 mL of methanol (CH_3_OH, Sigma-Aldrich) and left overnight. Then 2.5 mL of DPPH solution (0.04 mg mL^−1^) was added to 0.5 mL of the PGFs-methanol solution. The colour of the mixture changed from dark purple to yellow. UV-visible spectroscopy (Specord 40, Analytik Jena AG, Germany) at 517 nm was used to measure the absorbance of DPPH radicals. The decrease in absorbance at this wavelength indicates the antioxidant capacity of the tested solutions.

The radical-scavenging activity of each sample was also determined using the ABTS assay. Briefly, dissolution products were prepared by immersing 1 mg of PGFs in 1 mL distilled water and left overnight. An ABTS radical cation (ABTS˙^+^) stock was prepared by mixing 7 mM ABTS diammonium salt with 5 mM of potassium persulfate (Acros organics) aqueous solution and allowing the mixture to react in the dark for 12–16 h at room temperature. 50 µL of the ABTS˙^+^ working solution was added to 950 µL of each dissolution products in the wells of a 48-well flat-bottom microplate. The reaction mixture was incubated for 15 min at 25 °C, and the decrease in absorbance at 734 nm was recorded with a microplate reader (Tecan Spark X, Switzerland).

The radical-scavenging activity (RSA,% inhibition) for DPPH and ABTS assay was calculated using the following formula:2
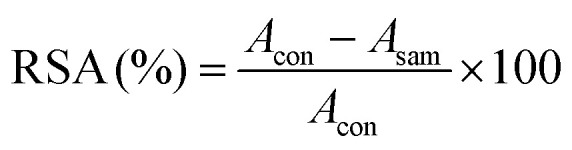
where *A*_con_ is the absorbance of the control and *A*_sam_ is the absorbance of the sample. For the DPPH assay, methanol was used as a blank, and the DPPH solution served as a control; for the ABTS assay, distilled water was used as a blank, and the ABTS˙^+^ solution served as a control. The DPPH and ABTS assays evaluate the antioxidant activity by measuring the reduction of radicals. In addition to DPPH and ABTS assays, the TMB assay was also performed to determine the ability to inhibit H_2_O_2_-derived radicals.

For the TMB assay, the Horseradish Peroxidase (HRP)/H_2_O_2_ system was used to oxidize colourless TMB into a blue-coloured radical cation (TMBox). The absorbance change of TMBox can reflect the OH˙ scavenging ability of the antioxidant. Briefly, a 1 mg mL^−1^ solution of the clv coated PGFs dissolution products (in DI water) was prepared and 50 µL of this sample was added to the solution containing 900 µL of NaAc-HAc buffer (mixing acetic acid and sodium acetate, pH 4.0), 1 mM of TMB (3,3′,5,5′-tetramethylbenzidine, 98%, TCI) solution, 1 mM H_2_O_2_, and 0.1 mM HRP (Dayang Demi) in a 48-well plate. After incubation at room temperature for 5–10 min, the reaction progress, driven by HRP-catalyzed oxidation of TMB to its blue radical cation. Change in absorbance was monitored spectrophotometrically at 652 nm. A decrease in absorbance indicates radical quenching by antioxidants released from PGFs. The experiment was conducted in triplicate (*n* = 3).

Antioxidant molecules (notably eugenol from clv) reduce the radical cation back to colourless TMB, diminishing the blue signal. Thus, the extent of absorbance reduction is proportional to the PGFs’ total antioxidant capacity.

### 2.8. Total phenolic content (TPC) assay of PGFs coated with clv

Different from the assays above, the TPC assay does not measure directly the radical scavenging capability but instead quantifies the total phenolic content of the sample. TPC of clv-coated PGFs was determined using the Folin–Ciocalteu assay (Sigma-Aldrich), following the protocol reported by Unalan *et al.*^31^ 1 mg of PGF was immersed in 1 mL of methanol for 24 h. 1 mL of Folin & Ciocalteu's phenol reagent solution (diluted 10 times with DI water) was then added to this solution. After 5 min, 2 mL of a 0.7 M sodium carbonate (Na_2_CO_3_, Sigma-Aldrich) (7.5 g sodium carbonate in 100 mL of DI water) were added to the mixture. Approximately 1.5 h later, the mixture changed from colourless to blue due to oxidation of phenols by tungstic/molybdic acid in the solution. Then UV-visible spectroscopy (Specord 40, Analytik Jena AG, Germany) was used to measure the total amount of phenolic compounds using the calibration curve of gallic acid (C_7_H_6_O_5_, GAE, Sigma-Aldrich) at 765 nm. GAE was used as a standard phenolic compound and DI water as a blank. The experiment was conducted in triplicate (*n* = 3).

### 2.9. Intracellular ROS assay in MC3T3–L1–E1 and HaCaTs

MC3T3–L1–E1 was seeded in 48-well plates at 5 × 10^4^ cells per well in Dulbecco's Modified Eagle Medium (DMEM, Gibco™) supplemented with 10% FBS and 1% Pen/Strep and allowed to adhere overnight at 37 °C in a humidified 5% CO_2_ atmosphere. PGF-Ga-clv3 (10 and 30 mg of PGFs in 10 mL of DI water) were incubated at 37 °C for 24 h, filtered through a 0.22-µm syringe-top filter (Millex™-GP), and used to prepare treatment media.

For preparation of the treatment media, an H_2_O_2_ working solution (100 µM) was prepared by diluting 3.6 µL of a 100 mM stock solution in DMEM to a final volume of 3.42 mL. 380 µL of this solution was combined with 20 µL of dissolution products (final volume 400 µL).

Untreated cells served as a negative control, and cells treated with H_2_O_2_ alone served as a positive control. After 4 h of exposure, the cells were washed twice with PBS and stained with 20 µM DCFH-DA for 30 min at 37 °C in the dark to detect ROS. They were then washed again with PBS and incubated with 5 µg mL^−1^ Hoechst for 10 min at room temperature. After washing the cells with PBS, oxidative stress and ROS scavenging by dissolution products were visualized using fluorescence microscopy (ZEISS Axio Observer, Germany). Fluorescence images were also analysed using Fiji, with green fluorescence intensity normalised to nuclei count. All experiments were performed in three technical replicates (*n* = 3).

In parallel, cell viability was assessed by seeding MC3T3–L1–E1 at 5 × 10^4^ cells per well in 48-well plates, incubating for 24 h, then treating for 4 h with 20 µL of each PGF-Ga-clv3 (1 mg mL^−1^ and 3 mg mL^−1^) dissolution products mixed with 380 µL of treatment media. Treatments were replaced with 10% MTT reagent in DMEM for 4 h at 37 °C, formazan was solubilized in 150 µL dimethyl sulfoxide (DMSO, 99.9%, Sigma-Aldrich) per well, and absorbance was read at 570 nm. Experiments were performed in three technical replicates (*n* = 3).

ROS generation was quantified in HaCaTs using the DCFDA/H2DCFDA Cellular ROS Assay Kit (ab113851, Abcam). This assay is based on the cell-permeant probe 2′,7′-dichlorofluorescin diacetate (DCFDA), which is deacetylated intracellularly and oxidized by ROS to yield the fluorescent compound DCF. Cells were seeded in 96-well plates and fluorescence was measured using a microplate reader at excitation/emission wavelengths of 485/535 nm. To assess the antioxidant activity under oxidative stress conditions, cells were exposed to 100 µM of *tert*-butyl hydroperoxide (TBHP) in the presence or absence of PGF-derived dissolution products. Under these conditions, the assay evaluates both basal ROS generation and the ability of the materials to suppress ROS formation during oxidative challenge. Experiments were performed in triplicate wells across three independents biological and technical replicates (*N* = 3, *n* = 3).

### 2.10. Wound closure *in vitro*

*In vitro* wound closure assessment was performed by creating a scratch to simulate an artificial wound in a confluent monolayer of cells (MC3T3–L1–E1 and HaCaTs) grown into a Petri dish and evaluating the scratch closure over time once in contact with PGFs’ dissolution products.

The dissolution products were prepared by immersing 10 mg of PGFs in 10 mL of DI water and incubated for 24 h at 37 °C at 200 rpm. Then, the suspensions were centrifuged at 4500 rpm for 5 min, and the supernatants were filtered using a 0.22 µm Millex™-GP filter unit.

#### 2.10.1. Scratch tests using MC3T3–L1–E1

MC3T3–L1–E1 was grown in DMEM supplemented with 10 vol% FBS and 1 vol% Pen/Strep. Cells were maintained at 37 °C in a humidified 5% CO_2_ incubator and sub-cultured at 70–80% confluence. MC3T3–L1–E1 were seeded in 24-well plates at 5 × 10^4^ cells per well and allowed to attach for 48 h. A linear scratch was introduced in each confluent monolayer with a sterile 200 µL pipette tip. For each condition, 20 µL of fibre extract were mixed with 380 µL of fresh DMEM containing FBS and Pen/Strep to give a working volume of 400 µL per well. Control wells received 400 µL of extract-free medium. Phase-contrast images were captured using optical microscopy (ZEISS Axio Observer, Germany) at 0, 6 and 24 h after scratching. The width of the scratch area was measured using the Fiji software^27^ and expressed as % of wound closure relative to the initial width. All experiments were performed with three technical replicates (*n* = 3).

#### 2.10.2. Scratch tests using HaCaTs

HaCaTs were seeded in 24-well plates and cultured to >95% confluence to form a uniform monolayer. To distinguish migration from proliferation, cells were pre-treated with mitomycin C (10 µg mL^−1^) for 2 h prior to scratching, thereby inhibiting cell division without significantly affecting viability. This ensures that wound closure reflects true migratory behaviour rather than proliferation, which is critical for long-term assays.

A linear scratch was introduced using a sterile pipette tip, and detached cells were removed by washing. The medium was then replaced with fresh medium containing either vehicle control or PGF-derived dissolution products. Images of the same marked regions were captured at 0, 24, 48, and 72 h using optical Nikon microscope. Wound closure was quantified by measuring the closure % using ImageJ software and expressed as a % of wound closure relative to the initial time point.

**Fig. 1:**
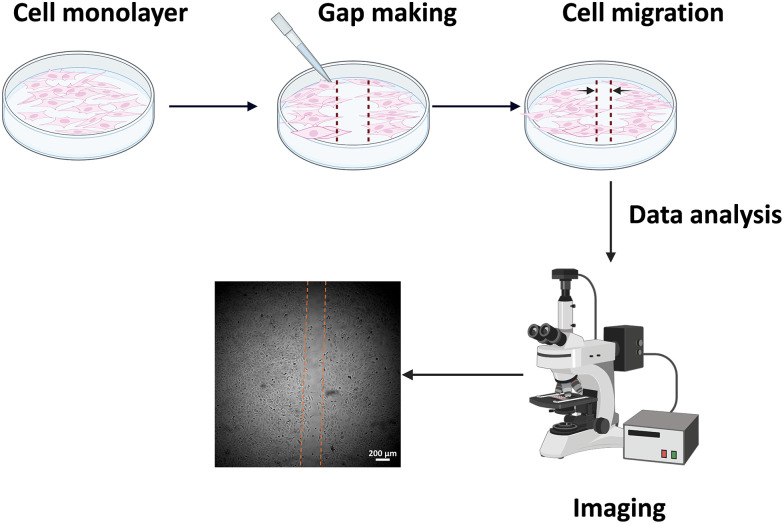
Schematic of the scratch test assay: a confluent monolayer is scratched to create a defined gap, cells migrate to close the wound, and gap closure is monitored by optical microscopy for quantitative analysis.

All experiments were performed with three biological and technical triplicates (*N* = 3, *n* = 3). Fig. 1 shows a schematic of the wound healing assay.

### 2.11. Statistical analysis

For antibacterial and all assays performed on MC3T3–L1–E1, data are presented as the mean ± standard deviation from at least three technical replicates (*n* = 3). For assays conducted on HaCaTs, data are presented as the mean ± standard error of the mean, based on three biological replicates with technical triplicates for each condition (*N* = 3, *n* = 3). GraphPad Prism software was used to perform all statistical analyses. The one-way and two way ANOVA with Dunnett's multiple comparison tests were performed for calculating statistically significant differences with *p ≤ 0.05 considered significant.

## 3. Results

### 3.1. Morphology and structure of PGFs

**Fig. 2:**
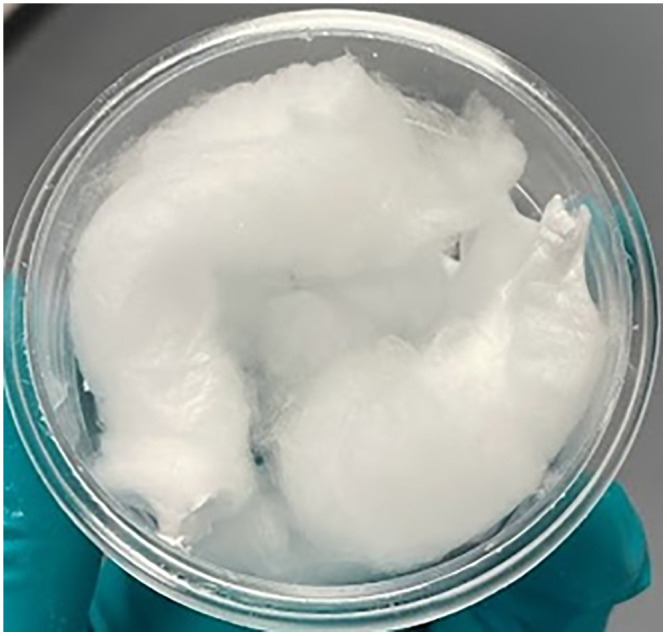
Optical image of a representative PGF demonstrating the cotton-like morphology.

All PGFs show a fluffy, cotton-like morphology (Fig. 2).

**Fig. 3:**
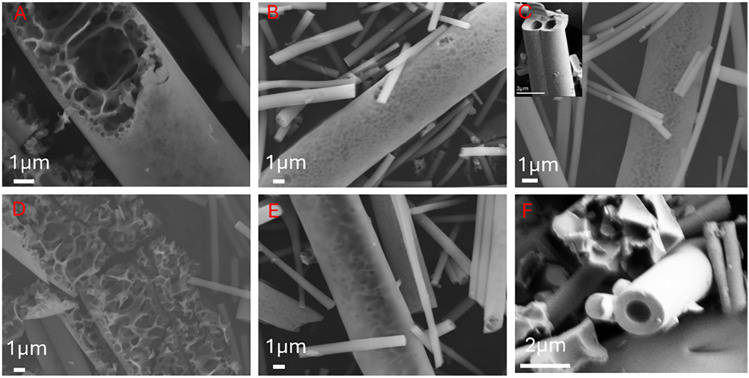
SEM images of (A) PGF-unl, (B) PGF-Ga0.2, (C) and (D) PGF-Ga0.5, (E) and (F) PGF-Ga1.

SEM images of PGF-unl, PGF-Ga0.2, PGF-Ga0.5 and PGF-Ga1 presented in Fig. 3A–E demonstrate that PGFs show extensive porosity, regardless of Ga loading. Fractured section of the ∼1–4 µm fibres (Fig. 3A and D) clearly shows sponge-like porosity within the PGFs’ walls with pores of size ∼300 nm. Pore shapes vary from round to slightly elongated. These pores arise from the removal of the CTAB micelles formed in water/ethanol, around which the inorganic precursor solidified.^33^ Interestingly, the smaller PGFs (average fibre diameter ∼0.8 µm) are hollow, with perfectly round channels with full walls (not porous) as evidenced in Fig. 3F and in the inset of Fig. 3C. The uniform central channel is ∼0.7 µm in diameter, which runs straight along the fibre axis, giving a true tubular shape, with uniform wall thickness (∼450 nm) (Fig. 3F).

**Fig. 4:**
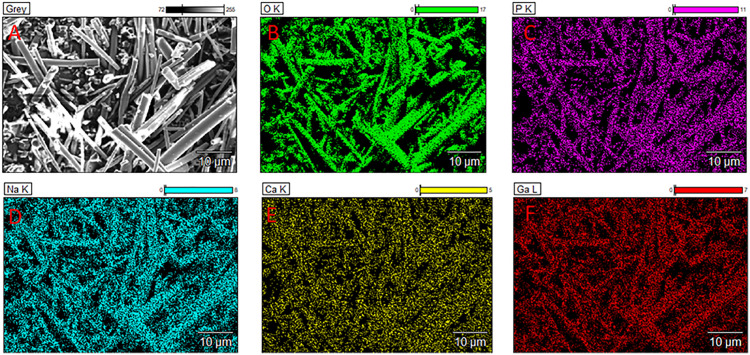
(A) SEM image of PGF-Ga1; (B) to (F) EDX elemental distribution maps of (B) O, (C) P, (D) Na, (E) Ca, and (F) Ga.

SEM combined with EDX were used to assess the composition of all PGFs. The EDX elemental mapping for the representative sample PGF-Ga1 is shown in Fig. 4. The SEM image (Fig. 4A, gray) shows the fibrous morphology; the porous structure is not clearly visible due to the low magnification. The elemental maps demonstrate that O (Fig. 4B, green), P (Fig. 4C, purple), Na (Fig. 4D, blue), Ca (Fig. 4E, yellow), and Ga (Fig. 4F, red) are evenly distributed on the surface of the fibres. Similar results have been obtained for all other PGFs. Overall, EDX analysis indicates compositional uniformity and chemical homogeneity of all PGFs.

Table 1 shows the elemental composition of all PGFs evaluated *via* EDX analysis. Data are expressed in both atomic% (at%) and oxide molar% (mol%). Oxide compositions shows that P_2_O_5_ is the major constituent (∼47–51%), followed by CaO (∼40–49 mol%) and Na_2_O (∼9–12 mol%). Ga_2_O_3_ content ranges from ∼0.2 to ∼0.8 mol%.

**Table 1:** Compositions of PGFs measured *via* EDX expressed as atomic% and oxide mol%. Values are reported as mean ± standard deviation (SD)

Sample	Atomic%					Oxide mol%			
	O	P	Ca	Na	Ga	P_2_O_5_	CaO	Na_2_O	Ga_2_O_3_
PGF-unl	65.0 ± 2.0	21.0 ± 1.0	8.7 ± 0.7	5.3 ± 0.1	—	47.7 ± 0.7	40.3 ± 1.0	12.0 ± 1.0	—
PGF-Ga0.2	61.2 ± 0.5	23.3 ± 0.5	10.8 ± 0.6	4.6 ± 0.4	0.10 ± 0.05	46.9 ± 0.2	43.9 ± 0.5	9.0 ± 1.0	0.2 ± 0.1
PGF-Ga0.5	62.0 ± 1.0	23.0 ± 1.0	9.0 ± 1.0	5.8 ± 0.3	0.20 ± 0.05	47.8 ± 0.5	40.1 ± 0.4	11.7 ± 0.4	0.4 ± 0.1
PGF-Ga1	59.0 ± 1.0	25.0 ± 1.0	11.0 ± 1.0	4.6 ± 0.3	0.40 ± 0.10	47.0 ± 0.7	43.6 ± 0.8	8.6 ± 0.7	0.8 ± 0.1

**Fig. 5:**
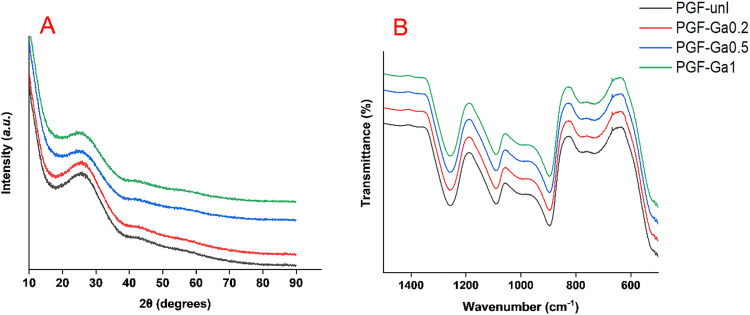
(A) XRD patterns and (B) FT-IR spectra of PGF-unl, PGF-Ga0.2, PGF-Ga0.5 and PGF-Ga1.

Structural analysis using XRD demonstrates that all PGFs are fully amorphous (Fig. 5A) with only a broad halo observed between 2*θ* ∼20 and 40° due to the phosphate network, rather than sharp diffraction peaks.^2^ FT-IR spectra of all PGFs (Fig. 5B) also exhibit the characteristic vibrations typical of amorphous phosphate networks. The bending *δ*(P–O–P) at ∼500–550 cm^−1^ and the symmetric and asymmetric stretching *ν*_s_ (P–O–P) and *ν*_as_ (P–O–P) at ∼750 and ∼900 cm^−1^, respectively arise from Q^2^ phosphate species. The stretching *ν*_s_ (PO_3_)^2−^ and *ν*_as_ (PO_3_)^2−^ modes at ∼1000 and ∼1100 cm^−1^ respectively, correspond to Q^1^ non-bridging oxygens terminal units (NBO). The band at ∼1180 cm^−1^ corresponds to the *ν*_s_ (PO_2_)^−^ symmetric stretching, while the band at ∼1270 cm^−1^ arises from the *ν*_as_ (PO_2_)^−^ asymmetric stretching of Q^2^ units (non-bridging out-of-chain). Overall, similar shapes and intensities across all compositions show that Ga does not significantly affect the phosphate network.^25^

### 3.2. Ion release

**Fig. 6:**
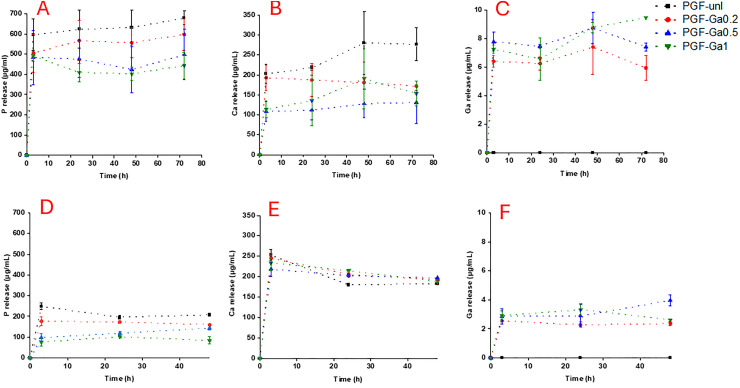
Release of (A) P, (B) Ca, (C) Ga in DI water over 72 h and (D) P, (E) Ca and (F) Ga in SBF over 48 h.

Fig. 6 shows the release of P, Ca and Ga after immersion of PGFs in DI water (Fig. 6A–C) and SBF (Fig. 6D–F) over 72 and 48 h, respectively.

In DI water, all species are released mostly in the first 3 h, regardless of the composition. P release (Fig. 6A) shows a clear decreasing trend with increasing Ga loading. P release from PGF-unl is ∼403 µg mL^−1^ and ∼507 µg mL^−1^ after 3 h and 72 h, respectively whereas P release from PGF-Ga1 is ∼353 µg mL^−1^ (3 h) and ∼354 µg mL^−1^ (72 h), respectively.

Release of Ca (Fig. 6B) is lower than that of P for all PGFs. Ca released from PGF-unl is ∼203 µg mL^−1^ after 3 h and ∼277 µg mL^−1^ after 72 h, whereas that released from PGF-Ga1 reached ∼113 µg mL^−1^ after 3 h and ∼153 µg mL^−1^ after 72 h.

Release of Ga (Fig. 6C) shows a clear compositional dependence. After 3 h, PGF-Ga0.2, PGF-Ga0.5 and PGF-Ga1 released ∼6, 7 and 8 µg mL^−1^ Ga respectively, rising only slightly by 72 h for PGF-Ga0.5 and PGF-Ga1 (∼7 and 10 µg mL^−1^ respectively).

Release of Na (Fig. SI_1) follows the same trend than P and Ca, with lower release from PGFs with higher Ga loading. The release of Na from PGF-unl reached ∼93 µg mL^−1^ after 3 h and increased to ∼112 µg mL^−1^ by 72 h.

Similarly to the results observed in DI water, most ions were released within the first 3 h when SBF was used as dissolution medium; however the amount of P and Ga released in SBF was lower than in DI water. Moreover, whilst in DI water different Ga loadings affect PGF's release, much more similar trends were observed in SBF. Release of Na could not be measured due to the high Na content in SBF.

P release (Fig. 6D) decreased with increasing Ga loading. P release from PGF-unl reached ∼248 µg mL^−1^ after 3 h and gradually decreased to ∼210 µg mL^−1^ after 48 h. In comparison, PGF-Ga1 released lower amounts of P, with values of ∼170 µg mL^−1^ after 3 h and ∼150 µg mL^−1^ after 72 h.

Ca release (Fig. 6E) followed a similar trend, with lower release observed for samples containing higher Ga levels after 3 h. Ca released from PGF-unl was ∼252 µg mL^−1^ after 3 h and gradually decreased to ∼187 µg mL^−1^ after 48 h. PGF-Ga1 exhibited lower Ca release, decreasing slightly from ∼233 µg mL^−1^ at 3 h to ∼187 µg mL^−1^ at 48 h.

Ga release from PGF-Ga0.2, PGF-Ga0.5 and PGF-Ga1 was approximately ∼2.5, ∼2.8 and ∼2.9 µg mL^−1^ after 3 h, respectively (Fig. 6F). Over 48 h, Ga release remained relatively stable for PGF-Ga0.2 and reached to ∼3.9 and ∼2.6 µg mL^−1^ for PGF-Ga0.5, and PGF-Ga1 after 48 h.

### 3.3. Release of clv from PGFs

**Fig. 7:**
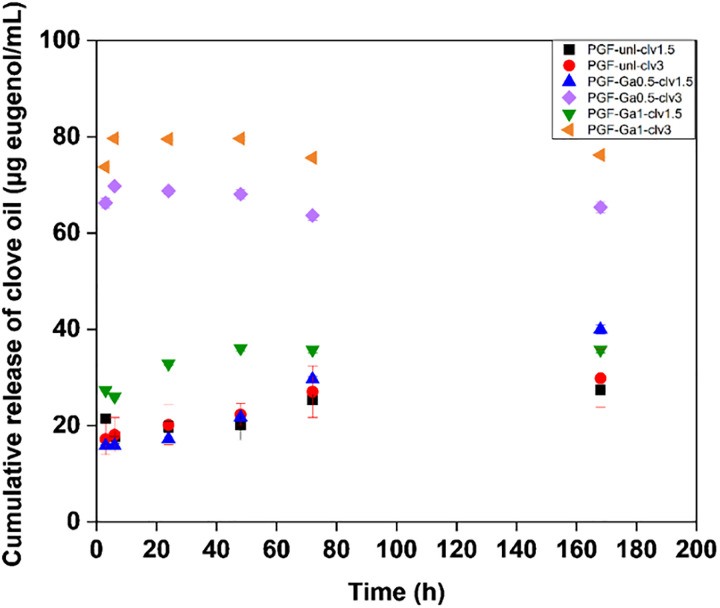
Cumulative release profile of clv from PGF-unl-clv1.5, PGF-unl-clv3, PGF-Ga.5- clv1.5, PGF-Ga.5-clv3, PGF-Ga1-clv1.5 and PGF-Ga1-clv3 in PBS at 37 °C. Values are given as mean ± standard deviation (*n* = 3).

To imbue PGFs with antioxidant activity, clv was added *via* impregnation at two concentrations (1.5 and 3 w/v%). Such relatively low concentrations of clv were chosen to minimise cytotoxicity risk, given that previous studies have demonstrated a dose-dependent cytotoxic effect in keratinocytes and fibroblasts, with higher concentrations of clv significantly reducing cell viability.^34^ Cumulative release profiles of clv from PGF-unl, PGF-Ga0.5 and PGF-Ga1 are presented in Fig. 7. PGF-unl exhibits an initial sharp release in the first 3 h, followed by gradual increase up to 168 h; for PGF-unl, no noticeable differences were observed between samples coated with different concentrations of clv, as both PGF-unl-clv1.5 and PGF-unl-clv3 exhibited very similar clv release. Results on PGF-Ga indicate that clv release depends both on the concentration of clv and Ga content. In particular, PGF-Ga0.5-clv1.5 and PGF-Ga1-clv1.5 have a lower clv release than PGF-Ga0.5-clv3 and PGF-Ga1-clv3. In addition, PGF-Ga0.5 has a lower clv release that PGF-Ga1, the highest clv release being from PGF-Ga 1-clv3. These findings are in agreement with our previous study on PGFs templated with P123 and suggest that Ga loading influences the release kinetics of clv, potentially due to the interactions between Ga and the clv molecules.^25^

### 3.4. HaCaTs viability

**Fig. 8:**
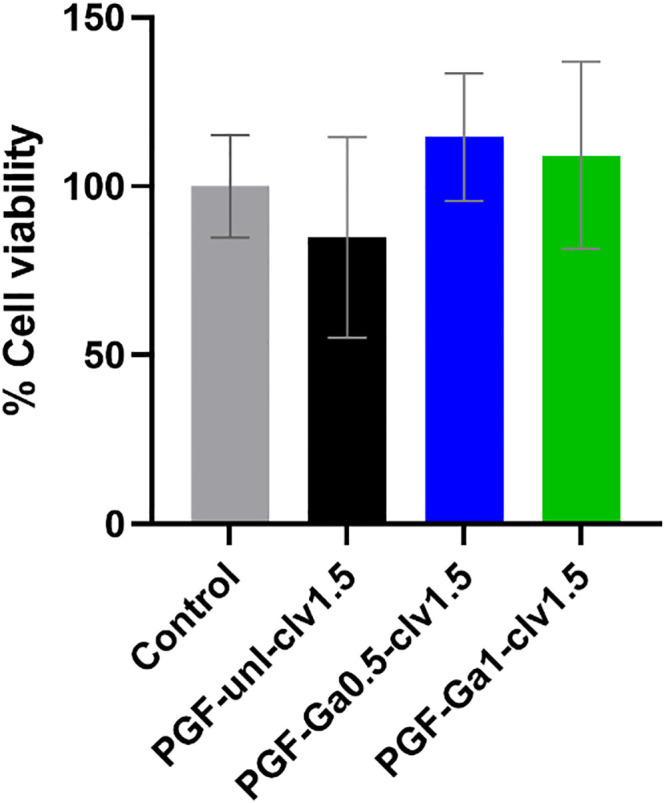
Viability of HaCaTs when exposed to PGF's dissolution products of PGF-unl-clv1.5 and PGFs-GaX-clv1.5 after 24 h in DI water (5% v/v). Error bars indicate the mean ± standard deviation (*n* = 3). Statistical analysis was performed using one-way ANOVA (*p ≤ 0.05). Asterisks illustrate the degree of statistical difference of the samples when compared to the control.

The viability of HaCaTs exposed to PGFs’ dissolution products of PGF-unl-clv1.5 and PGF-Ga-clv1.5 was evaluated after 24 h (Fig. 8). For samples coated with 1.5 w/v% clv, Ga-free sample exhibited a cell viability of 85% and Ga-containing samples showing values close to 100%, indicating no cytotoxic effects for PGF-clv1.5.

**Fig. 9:**
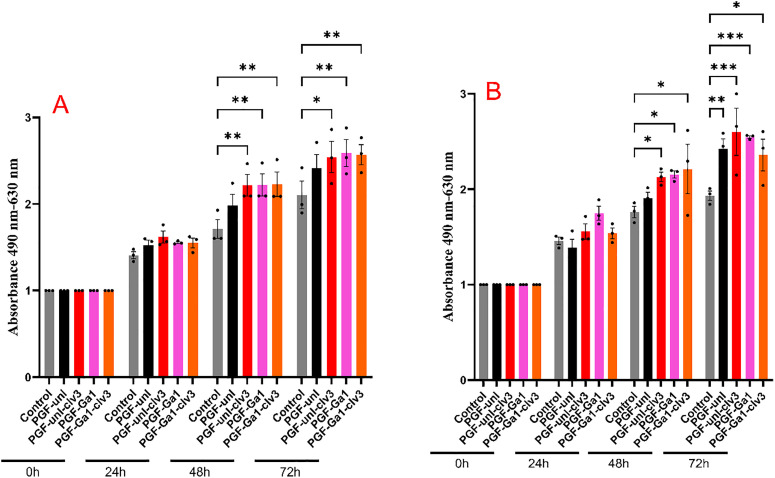
Viability of HaCaTs when exposed to PGF's dissolution products (over 72 h immersion) for two ratios dissolution products/medium (A) 20 µL in 380 µL of medium, (B) 40 µL in 360 µL of medium. Data are mean ± standard error of the mean (*N* = 3 biological replicates; each averaged from three technical replicates *n* = 3; two-way ANOVA with Dunnett's multiple comparisons).

To further assess potential time-dependent effects, HaCaTs viability was evaluated over 72 h using the MTS assay (Fig. 9). Across all timepoints (0, 24, 48, and 72 h) and for both extract concentrations (5% and 10% v/v), cell metabolic activity remained stable, with no reduction compared to the untreated control. Notably, a gradual increase in metabolic activity was observed over time in all groups, consistent with normal cell proliferation. Treated samples exhibited comparable or slightly higher metabolic activity than the control at later timepoints, indicating that the dissolution products do not induce delayed cytotoxic effects and may support cell viability under these conditions.

This trend was consistent across both concentrations, demonstrating that increasing extract content within the tested range does not adversely affect cytocompatibility. The inclusion of the 0 h timepoint further confirms uniform initial cell seeding, and consistent results obtained using both normalized and raw data indicate that the measurements are not influenced by the presence of dissolution products.

### 3.5. Antibacterial activity

**Fig. 10:**
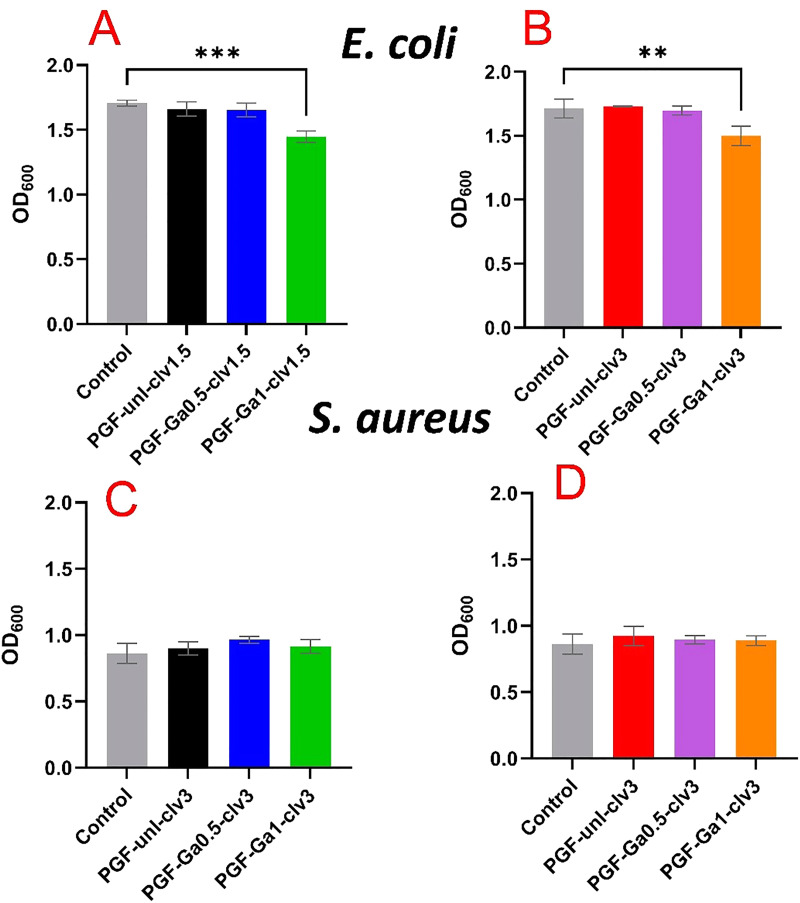
Antibacterial activity of dissolution products of (A) PGF-Ga-clv1.5 and (B) PGF-Ga-clv3 against *E. coli* and (C) PGF-Ga-clv1.5 and (D) PGF-Ga-clv3 against *S. aureus* after 24 h immersion in DI water. Error bars indicate the mean ± standard deviation (*n* = 3). Statistical analysis was performed using one-way ANOVA (**p ≤ 0.01, and ***p ≤ 0.001). Asterisks illustrate the degree of statistical difference of samples compared to the control.

As Ga ions are known for their antibacterial activity, the effect of PGFs’ dissolution products on the growth of *E. coli* and *S. aureus* was investigated by measuring OD_600_. Fig. 10A and B show *E. coli* growth (measured as OD_600_) after 24 h in the presence of 1.5 and 3 w/v% clv coated PGFs with different Ga loadings or TSB alone as a control, respectively. At both clv concentration (1.5 or 3 w/v%), PGF-unl-clv and PGF-Ga0.5-clv exposure had no significant impact on *E. coli* growth, while PGF-Ga1-clv1.5 and PGF-Ga1-clv3 resulted in statistically significant reduction in OD_600_. In contrast, exposure of *S. aureus* to dissolution products (Fig. 10C and D) was observed to have no effect on bacterial growth across all PGFs for both clv concentrations (1.5 and 3 w/v% clv). Collectively, these results demonstrate that PGFs with the highest Ga concentration (1 mol%) produce a significant reduction in bacterial growth, but this effect may differ based on bacterial species.

**Fig. 11:**
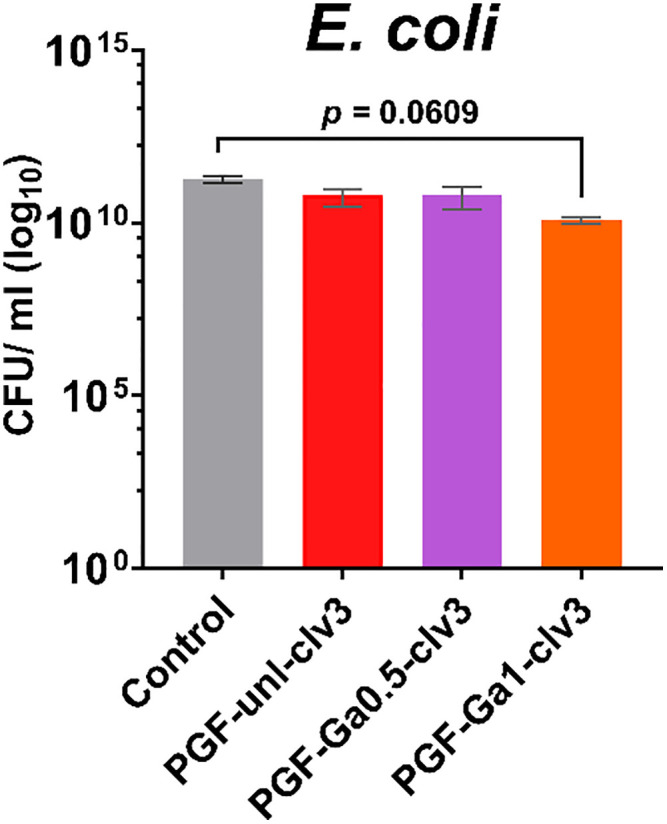
Bacterial viability of *E. coli* following incubation of dissolution products of PGF-unl-clv3 and PGF-GaX-clv3 for 24 h in TSB. Error bars indicate the mean ± standard deviation (*n* = 4). Statistical analysis was performed using one-way ANOVA and Dunnett's test for comparison to the control.

To further elucidate the dynamics of the observed antibacterial activity  of the PGFs’ dissolution products against *E. coli*, bacterial viability was quantified following culturing of *E. coli* with 3 w/v% clv coated PGFs. Similarly to what was observed in the OD_600_ assay, PGF-unl-clv3 and PGF-Ga0.5-clv3 exposure demonstrated no antibacterial activity, with no significant changes in bacterial viability (Fig. 11). In contrast, exposure of PGF-Ga1-clv3 to *E. coli* resulted in a reduction in bacterial viability following incubation, but this was not statistically significant, suggesting that the effect of PGFs’ dissolution products may be bacteriostatic opposed to bactericidal.

### 3.6. Antioxidant assays

**Fig. 12:**
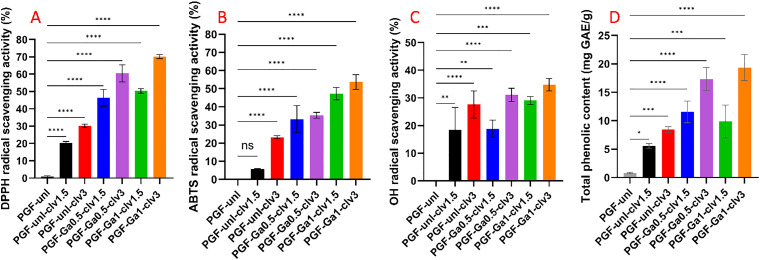
(A) DPPH radical scavenging activity, (B) ABTS assay, (C) OH˙ scavenging using TMB assay and (D) TPC assay for PGF-unl, PGF-unl-clv, PGF-Ga0.5-clv, PGF-Ga1-clv loaded with 1.5 and 3 w/v% clv. Error bars indicate the mean ± standard deviation (*n* = 3). Statistical analysis was performed using one-way ANOVA (*p ≤ 0.05, **p ≤ 0.01, ***p ≤ 0.001 and ****p ≤ 0.0001). Asterisks illustrate the degree of statistical difference of the samples when compared to the control.

Antioxidant properties were investigated on PGF-Ga0.5 and PGF-Ga1 coated with clv (1.5 and 3 w/v%) and on PGF-unl as a control to investigate both the effect of Ga and clv and possible synergistic effects. Complementary antioxidant assays were performed: DPPH (Fig. 12A), ABTS (Fig. 12B), TMB (Fig. 12C) and TPC (Fig. 12D).

Antioxidant assays demonstrate that the incorporation of clv and Ga markedly enhances the antioxidant potential of PGFs. The PGF-unl showed negligible antioxidant activity, with DPPH, ABTS, and OH˙ scavenging values close to zero. Upon loading 1.5 w/v% clv (PGF-unl-clv1.5), these values increased substantially to 20.0 ± 0.8% for DPPH, 6.0 ± 0.2% for ABTS, and 18 ± 6% for OH˙, accompanied by a rise in TPC to 5.0 ± 0.4 mg GAE g^−1^, indicating that the phenolic groups from clv significantly contribute to radical scavenging through hydrogen or electron donation. By doubling the clv concentration to 3 w/v% (PGF-und-clv3), a further enhancement of antioxidant performance was observed, with DPPH, ABTS, and OH˙ scavenging values reaching 30 ± 1%, 24.0 ± 0.8%, and 25 ± 4%, respectively, and a corresponding increase in TPC to 8.0 ± 0.5 mg GAE g^−1^. This clear concentration dependent trend reflects the direct relationship between phenolic content and free radical neutralization capacity.^35^ The addition of Ga further enhances these effects, suggesting an additive effect between Ga ions and phenolic compounds. For PGF-Ga0.5-clv1.5, the DPPH and ABTS scavenging activities rose to 45 ± 5% and 33 ± 7%, respectively, while OH˙ scavenging remained at 18 ± 3%, with TPC increasing to 11 ± 2 mg GAE g^−1^. The highest antioxidant activity was observed for PGF-Ga1-clv3, indicating that increased Ga and clv concentrations could enhance antioxidant performance, with DPPH, ABTS, and OH˙ scavenging reaching 70 ± 2%, 53 ± 4%, and 34 ± 2%, respectively, and TPC peaking at 18 ± 2 mg GAE g^−1^. These results suggest that the electron-transfer-based DPPH and ABTS assays are more sensitive to the phenolic-Ga interactions than the TMB assay, which relies on direct radical OH˙ quenching. Overall, the parallel increase in TPC and radical scavenging efficiencies confirms that higher phenolic content, enhanced by Ga loading, strengthens the antioxidant capacity of PGF-Ga-clv.

### 3.7. Intracellular ROS Assay in MC3T3–L1–E1 and HaCaTs

Oxidative stress often occurs when the production of ROS exceeds the capacity of the cell's antioxidant defence systems to neutralise them. This imbalance leads to the accumulation of ROS such as superoxide, hydrogen peroxide, and hydroxyl radicals, which can damage lipids, proteins, and DNA.^36^ To determine if the PGF-Ga-clv system could mitigate this damage and support tissue regeneration, ROS assays were conducted on both MC3T3–L1–E1 and HaCaTs under chemically induced oxidative stress using two different assays.

**Fig. 13:**
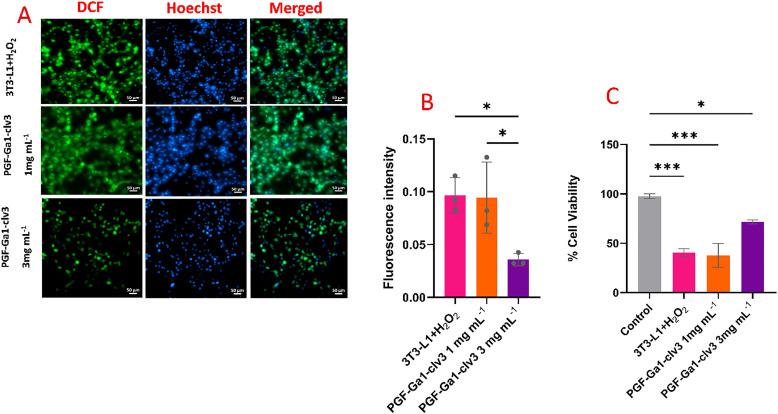
ROS detection: (A) fluorescence micrographs of H_2_O_2_ treated- MC3T3–L1–E1 stained with DCFH-DA (left column panes, green, ROS indicator) and stained with Hoechst (central column panes, blue, nuclei) and merged images (right column panes) after exposure to PGF-Ga1-clv3, 1 mg mL^−1^, and PGF-Ga1-clv3 3 mg mL^−1^; (B) normalised intracellular ROS fluorescence intensity in H_2_O_2_-treated cells with or without PGF-Ga1-clv3 (1 and 3 mg mL^−1^) (C) Cell viability of MC3T3–L1–E1 following treatment in the same conditions. Untreated cells (negative control) and H_2_O_2_-treated cells (positive control) were used as normal and oxidative stress controls, respectively. Values are given as mean ± standard deviation (*n* = 3). The asterisks indicate a statistically significant difference (*p ≤ 0.05, and ***p ≤ 0.001).

In the initial assay involving MC3T3–L1–E1, the antioxidant properties of the PGF-Ga1-clv3 dissolution products were evaluated at two concentrations: 1 mg mL^−1^ and 3 mg mL^−1^. These concentrations were achieved by immersing 10 mg and 30 mg of PGFs in 10 mL of DI water, respectively. To induce oxidative stress, all cells were first exposed to H_2_O_2_ and then treated with either the PGF-Ga1-clv3 dissolution product at 1 mg mL^−1^ or 3 mg mL^−1^, or left untreated as a positive control. Fluorescence micrographs illustrating intracellular ROS levels under each treatment condition are shown in Fig. 13A: H_2_O_2_-treated cells with no further treatment (positive control), and H_2_O_2_-treated cells subsequently exposed to PGF-Ga1-clv3 at 1 mg mL^−1^ or 3 mg mL^−1^.

The antioxidant effect was assessed by measuring the reduction of intracellular ROS using DCFH-DA fluorescence. Inside the cells, DCFH-DA is de-acetylated to non-fluorescent DCFH, which in turn is oxidised by ROS to yield the bright green fluorophore DCF (2′,7′-dichlorofluorescein) (Fig. 13A, left column panes, green). Fluorescence intensity serves as a semiquantitative indicator of overall oxidative load. Therefore, the brighter the green colour is, the more ROS are present. Nuclei were visualised using Hoechst staining (Fig. 13A, central column panes, blue) to evaluate cell density and nuclear morphology. The corresponding merged images are shown in the right column panes of Fig. 13A. The positive control shows a diffuse green fluorescence throughout the cytoplasm and around the nuclei, indicating strong oxidative stress following H_2_O_2_ exposure. As shown in Fig. 13B, cells treated with the H_2_O_2_ and PGF-Ga1-clv3 dissolution product at 1 mg mL^−1^ retained a DCF fluorescence intensity comparable to the positive control, indicating that the antioxidant capacity at this concentration was insufficient to significantly neutralise the incoming ROS flux. However, when the H_2_O_2_-treated cells were treated with the PGF-Ga1-clv dissolution product (3 mg mL^−1^) a significant decrease of DCF fluorescence was observed. Most of MC3T3–L1–E1 exhibited only weak green fluorescence. Because the DCFH-DA assay integrates multiple ROS types-superoxide, hydroxyl radical, and peroxides, this reduction shows broad-spectrum scavenging activity.

Cell viability was assessed using the MTT assay following the same treatment conditions (Fig. 13C). Untreated cells not exposed to H_2_O_2_ served as the negative control.

Cell viability fell to 40 ± 4% after H_2_O_2_ treatment and a similar reduction was observed when cells treated with H_2_O_2_ and PGF-Ga1-clv dissolution product (1 mg mL^−1^). However, a cell viability of 70 ± 2% was observed when H_2_O_2_-treated cells received PGF-Ga1-clv dissolution product (3 mg mL^−1^). This agreement shows that high ROS levels can decrease mitochondrial enzymes needed to convert MTT, while lowering ROS keeps those enzymes working and helps cells stay alive. Overall, the fluorescence images and the viability assays confirm that the PGF-Ga1-clv (3 mg mL^−1^) dissolution products give dose-dependent antioxidant protection inside MC3T3–L1–E1 facing acute oxidative stress.

**Fig. 14:**
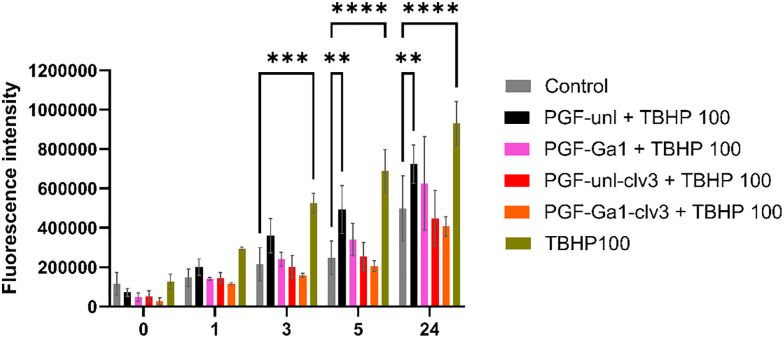
Intracellular ROS response in HaCaTs over time under TBHP-induced oxidative conditions. Data are presented as mean ± standard error of the mean (*N* = 3 biological replicates), where each biological replicate represents the average of three technical replicates (*n* = 3). Statistical analysis was performed using two-way ANOVA (Dannett's multiple comparison test). A *p*-value < 0.05 was considered statistically significant *p*-values: **p < 0.01; ***p < 0.001, ****p < 0.0001. Asterisks illustrate the degree of statistical difference of the samples when compared to the control.

The ability of PGFs’ dissolution products to mitigate oxidative stress was further evaluated in HaCaTs exposed to TBHP (100 µM), a potent pro-oxidant. As shown in Fig. 14, the TBHP-only control induced a significant increase in fluorescence intensity across all time points, confirming effective oxidative stress induction. In contrast, all groups treated with PGFs’ dissolution products exhibited reduced ROS levels compared to the TBHP control throughout the duration of the assay, indicating a protective antioxidant effect.

Statistical analysis revealed that while PGF-unl provided a baseline level of ROS reduction, the incorporation of Ga and clv significantly enhanced the antioxidant response. At early time points (3 and 5 h), PGF-Ga1 and PGF-unl-clv3 showed significant reductions in ROS levels compared to the TBHP control.

The strongest effect was observed for PGF-Ga1-clv3, which consistently exhibited the lowest fluorescence intensity across all time points. Notably, by 24 h, ROS levels in PGF-unl-clv3 and PGF-Ga1-clv3 were comparable to the untreated control, indicating sustained suppression of oxidative stress under pro-oxidant conditions.

### 3.8. Wound healing scratch assay

**Fig. 15:**
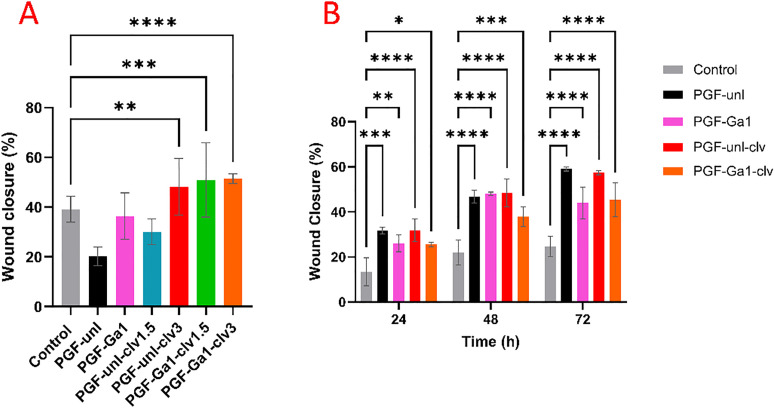
*In vitro* monolayer scratch assay of (A) untreated MC3T3–L1–E1 (control) and those treated with PGF-unl, PGF-Ga1, PGF-unl-clv1.5, PGF-Ga1-clv1.5, PGF-unl-clv3, PGF-Ga1-clv3 after 6h; (B) untreated HaCaTs (control) and those treated with PGF-unl, PGF-Ga1, PGF-unl-clv3 and PGF-Ga1-clv3 after 24, 48 and 72 h. The asterisks indicate a statistically significant difference (**p ≤ 0.01, ***p ≤ 0.001 and ****p ≤ 0.0001).

The *in vitro* scratch assay for assessing wound closure is a simple tool for modelling how a cell monolayer responds to injury in the presence of a test material. A straight “scratch” is made through a confluent monolayer, creating a cell-free gap that mimics a wound. By recording the gap width at defined intervals, it is possible to quantify the time required for complete closure and monitor the dynamic processes of cell migration and proliferation that drive healing.^37^Fig. 15 shows the scratch assay results for MC3T3–L1–E1 and HaCaTs monolayers, respectively treated with control, and dissolution products from PGF unloaded, coated with 1.5 and 3 w/v% clv.

Both cell lines are commonly used as biologically relevant model for assessing wound closure, MC3T3–L1–E1 as a fibroblast cell line^38–40^ and HaCaTs (keratinocytes) due to their central role in re-epithelialisation.^41^

The scratch assay images for PGF-unl, PGF-Ga, PGF-unl-clv, and PGF-Ga-clv on MC3T3–L1–E1 and HaCaTs are presented in Fig. SI_2 and SI_3, respectively with the region between the red square representing the “artificial wound” created by the scratch.

When using MC3T3–L1–E1, under standard culture conditions (Fig. 15 A), at 0 h the width of the scratch for the control monolayer shows is ∼650 µm. By 6 h the gap was ∼393 µm wide (39 ± 5% closure), and by 24 h it was completely closed. Thus, under standard culture conditions the cell line achieves 100% closure within 24 h. For PGF-unl and PGF-Ga1 the initial scratch widths were ∼882 µm and ∼713 µm, respectively. For PGF-unl a 20 ± 4% closure was observed after 6 h (∼702 µm) and full closure at 24 h. For PGF-Ga1, the closure at 6 h occurred faster than for PGF-unl, around 33 ± 9% (∼450 µm) and full closure was reached at 24 h.

The widths of the scratchs in PGF-unl-clv1.5 and PGF-Ga1-clv1.5 at 0 h were 614 µm and 588 µm, respectively. Both PGF-unl-clv 1.5 and PGF-Ga1-clv 1.5 show higher closure% than for PGF-unl and PGF-Ga1. PGF-unl-clv1.5 shows approximately 30 ± 5% (∼428 µm) and PGF-Ga1-clv 1.5 shows around 50 ± 14% (∼291 µm) closure. The scratch widths for PGF-unl-clv3 and PGF-Ga1-clv3 were ∼632 µm and ∼545 µm, respectively. After 6 h, the wounds treated with PGF-unl-clv3 and PGF-Ga1-clv3 showed approximately 48 ± 11% (∼324 µm) and 51 ± 2% (∼264 µm) closure. Table SI_1 shows the scratch width and percentage of wound closure for MC3T3–L1–E1 in contact with PGFs dissolution products. This indicates that adding Ga and clv accelerates early cell migration.

The wound closure effect of selected PGFs’ dissolution products was further evaluated in HaCaTs over extended timepoints (24, 48, and 72 h) (Fig. 15B).

Unlike the scratch assays performed on MC3T3–L1–E1, HaCaTs assays were conducted following mitomycin C treatment (10 µg mL^−1^, 2 h) to inhibit proliferation. This ensures that wound closure reflects true cell migration rather than proliferation, which is particularly important for long-term assays given the relatively short doubling time of HaCaTs.

Wound closure was observed across all treatment groups, with PGFs’ dissolution products consistently enhancing cell migration compared to the untreated control. At 24 h, the control exhibited minimal closure (∼13%), whereas all treated groups showed significantly increased migration (∼25–35%). By 48 h, differences between groups became more pronounced. PGF-unl and PGF-Ga1 demonstrated enhanced wound closure (∼47%), significantly higher than the control (∼20%), while PGF-unl-clv3 showed comparable activity (∼49%). PGF-Ga1-clv3 exhibited a slightly lower, yet still enhanced, response (∼38%) at this intermediate time point.

At 72 h, wound closure reached maximal levels across all groups, with PGF-unl and PGF-unl-clv3 achieving the highest closure (∼58%). Overall, these results demonstrate that PGFs’ dissolution products do not impair, and can enhance cell migration in HaCaTs, confirming that the release of Ga and clv does not adversely affect cellular behaviour and positively affect wound closure.

## 4. Discussion

Porous PGFs were successfully prepared *via* ES of coacervate gels containing the templating agent CTAB. Structural analysis by XRD and FT-IR confirmed their amorphous nature, with a network structure similar to powder PGs and non-porous PGFs.^2,42^ Interestingly, SEM analysis revealed that after calcination at 350 °C, CTAB-templated PGFs are all hollow with a distinctive wall morphology depending on their size: porous walls for bigger fibres and uniform walls for smaller ones. Fibres with diameters of ∼1–4 µm exhibited highly porous walls with pores of ∼300 nm in diameter, whereas a smaller set of fibres with an average diameter of ∼0.8 µm displayed well-defined cylindrical shapes with dense walls. Formation of hollow fibres is consistent with previous CTAB-assisted ES manufacturing of hierarchical hollow magnesium oxide nanofibers.^43^ This morphology can be attributed to the combination of phase separation and solvent evaporation kinetics during electrospinning. For larger fibres, slower solvent evaporation allows sufficient time for CTAB micelle templating and phase separation throughout the fibre cross-section, resulting in sponge-like porous walls after micelle removal. In contrast, smaller fibres experience much faster solvent evaporation due to their higher surface-area-to-volume ratio. This may be due to a limited phase separation within the fibres, which promotes the formation of a central hollow region, resulting in fibres with dense, non-porous walls.^44^

Hollow and tubular silicate-based glass fibres have been reported to enhance bioactivity, drug loading capabilities^45^ and proliferation rate of mouse pre-osteoblastic MC3T3–L1–E1 cells compared to the corresponding bulk fibres.^46^ As the fibrous porous morphology is ideal for wound repair and the presence of porosity and channels in PGFs is desirable for enhanced loading of therapeutic species, the release of therapeutic ions and clv, which could have a beneficial effect on wound healing (*e.g.* antibacterial, antioxidant), was investigated.

Among the therapeutic ions, Ca^2+^ plays a crucial role in wound healing by supporting haemostasis (coagulation) and acting as a key signalling cue that regulates keratinocyte and fibroblast behaviour (proliferation, differentiation, and migration), thereby promoting re-epithelialisation and tissue remodelling.^33,47,48^ Ga^3+^ has also been reported to exhibit potent haemostatic activity, enhancing intrinsic coagulation, thrombus formation, and platelet adhesion/activation^49^ while providing antibacterial efficacy by disrupting bacterial iron metabolism, which supports re-epithelialisation, angiogenesis, and tissue regeneration and accelerating wound closure.^50^ In addition, phosphate anions can contribute to repair by acting as a metabolic energy source that increases extracellular ATP levels, helping meet the high energetic demand of processes such as angiogenesis.^51^

Release profiles in DI water indicate that Ga release increases with increasing Ga content reaching a maximum of ∼10 µg mL^−1^ for PGF-Ga1 after 72 h. Calcium release is also compositional-dependent as the highest value is observed for the Ga-free fibres with a maximum value of 276 µg mL^−1^ after 72 h. Similarly, the release of P decreases with increasing Ga content, suggesting that Ga ions act as network cross-linkers. This behaviour is in agreement with previous findings where the formation of strong Ga–O–P bonds was reported, with an increase in network connectivity and reduction of phosphate depolymerisation.^25,52^ These results indicate the versatility of the systems as ion release can be controlled by tailoring the Ga content in the PGFs.

Ion release in SBF has revealed smaller differences between samples with different Ga loadings, and lower P and Ga amount released over the same timeframe than in DI water. Since SBF is a solution designed to mimic the ionic concentration of blood plasma, the concentration gradient between the glass surface and the surrounding fluid is much lower than in DI water, which inhibits the rapid release of ions from the glasses.^53^

PGFs coated with the natural antioxidant clv also showed a controlled release over time with the highest values of clv released registered from PGF-Ga0.5-clv3 and PGF-Ga1-clv3 (∼68 and ∼79 µg mL^−1^ after 24 h, respectively). This is of particular interest for the release of antioxidant species such as clv, which play a crucial role in the wound healing process by ensuring the correct balance of ROS.^54^

Biocompatibility testing in HaCaTs demonstrated that clv loading influences cell viability in a concentration-dependent manner. At 24 h, all PGFs coated with 1.5 w/v% clv exhibited viability values close to 100%, indicating excellent cytocompatibility. Importantly, time-dependent MTS assays revealed that HaCaTs viability was maintained over 72 h across all tested conditions, including PGF-unl, PGF-Ga1, PGF-unl-clv3, and PGF-Ga1-clv3, with sustained metabolic activity observed throughout the study period. These results indicate the absence of delayed cytotoxic effects and confirm the overall cytocompatibility of the materials. These findings are consistent with previous reports on mesoporous bioactive glass nanoparticles coated with clv, where cell viability values above 80% were observed in MG-63 cells.^21^ It has to be noted that eugenol/phenolic compounds at high concentration could cause some toxicity.^55^

To validate the multifunctionality of PGFs, the antibacterial effect of Ga and clv on the common strains *E. coli* and *S. aureus* was also investigated, along with exploration of additive effects. Ga has been reported to have antibacterial effects against *E. coli* on porous PGFs templated with P123^25^ and in MQ quaternary Ga-doped PGs powders containing 1 mol% Ga_2_O_3_.^56^ Clv has also been widely reported to exhibit antibacterial activity. Unalan *et al.* demonstrated that clv-loaded poly(ε-caprolactone)/gelatin electrospun nanofiber mats significantly reduced the viability of *S. aureus* and *E. coli*, indicating broad-spectrum antibacterial efficacy compared with clv-free mats.^57^ Our results indicate that for the concentrations used, the antibacterial activity against *E. coli* is ascribed to Ga rather than to clv. A dose-dependent effect was observed, with 1 mol% identified as the concentration of Ga needed to produce an effect, as lower loading (0.5 mol%) gave results comparable to the control. The lack of inhibitory effects from clv may be attributed to the amount released that is unable to produce an effect. The antibacterial findings were further investigated by CFU analysis, which confirmed a reduction in viable bacterial counts for PGF-Ga1-clv3. Furthermore, the dissolution products did not exhibit antibacterial activity against *S. aureus*, despite the incorporation of Ga and clv, suggesting a limited effectiveness of these components against this strain. These results are in agreement with previous work that suggests that *S. aureus* has a much stronger anti-gallium defence system than *E. coli*.^25,58^

*S. aureus* cells have a thicker cell wall and a noticeable nucleoid housing DNA molecule, in comparison to *E. coli* cells. After treatment with Ga^3+^, *E. coli* cells displayed damage and indistinctness in certain parts of the cell wall, whereas *S. aureus* cells maintained an intact cell structure without any observed broken segments. This suggests a stronger defence mechanism against Ga in *S. aureus*.^58^ In contrast to the antibacterial behaviour, all PGF-Ga containing clv showed strong antioxidant activity. Clv has a clear effect on its own, as by increasing the loading from 1.5 to 3 w/v% in Ga-free samples, the antioxidant activity increases. In addition, the activity increases also by increasing the Ga content only and maintaining constant clv loading. Therefore, there is an additive effect of clv and Ga when both are simultaneously used. Overall, DPPH, ABTS, TMB, and TPC results indicate a broad-spectrum of antioxidant activities, including free-radical scavenging, electron-donating capacity, and phenolic-based antioxidant potential.

The biological relevance of the antioxidant behaviour was further investigated through intracellular ROS assays in both MC3T3-L1-E1 and HaCaTs. While chemical assays (DPPH, ABTS, TMB) demonstrate radical scavenging capacity in solution, the cellular ROS results confirm that these materials can effectively mitigate oxidative stress in a biological environment. In particular, PGF-Ga1-clv3 showed a pronounced reduction in ROS levels under oxidative challenge (H_2_O_2_ or TBHP) and reducing oxidative stress.

As the results demonstrated, the MTS assay showed that the PGF dissolution products did not induce cytotoxic effects, indicating good cytocompatibility across all tested conditions. These findings are further supported by the ROS assay, which was performed under TBHP-induced oxidative stress. Where the dissolution products not only avoided inducing ROS generation but also significantly reduced ROS levels in the presence of a pro-oxidant stimulus. Together, these results indicate that the materials are not intrinsically pro-oxidative and, importantly, exhibit a protective effect by mitigating ROS generation under oxidative stress conditions. Importantly, the wound closure assay demonstrated enhanced cell migration in response to Ga- and clv-loaded PGFs, with up to ∼51% closure observed in MC3T3-L1-E1 after 6 h and up to ∼58% closure in HaCaTs after 72 h. These results indicate a consistent pro-migratory effect of the dissolution products across different cell types. As expected, fibroblasts exhibited a faster rate of wound closure compared to keratinocytes, reflecting their intrinsic migratory behaviour and shorter response time in early-stage wound healing.^59^

While previous studies on porous Ga/clv-loaded PGFs have primarily focused on material synthesis, release behaviour and antioxidant characterisation, the present work extends the evaluation to biologically relevant wound-healing models. The combined assessment of long-term keratinocyte viability, oxidative stress modulation under pro-oxidant conditions, antibacterial activity and migration in both fibroblast-like and keratinocyte cell lines provides a more comprehensive understanding of how these materials interact with cellular processes relevant to wound repair.

Importantly, the results demonstrate that the materials are not only non-cytotoxic over extended exposure periods but are also capable of suppressing oxidative stress and promoting cell migration. Together, these findings establish a stronger biological rationale for the use of porous Ga/clv-loaded PGFs in wound-healing applications and significantly expand upon previous reports that were primarily focused on physicochemical and antioxidant properties.

The *in vitro* scratch assay used to assess wound closure has inherent limitations, as it is performed on a two-dimensional monolayer of cultured cells. In contrast, the *in vivo* wound healing process is considerably more complex, involving coordinated phases of inflammation, cell proliferation, extracellular matrix remodelling, and angiogenesis. Therefore, while the present results provide valuable insight into the pro-migratory properties and cytocompatibility of the PGFs, *in vivo* studies will be required to fully evaluate their wound healing potential. WVTR measurements will be also needed for assessment of optimal moisture balance. Results have demonstrated that porous PGF-Ga-clv presented in this work have great potential to be used as multifunctional materials for wound healing by combining biocompatibility, antibacterial activity, strong antioxidant performance and wound closure.

## 5. Conclusions

This work demonstrates the successful synthesis of porous PGFs in the P_2_O_5_–CaO–Na_2_O–Ga_2_O_3_ system (Ga_2_O_3_: 0.2, 0.5 and 1 mol%) by ES of a coacervate polyphosphate gel templated with CTAB. Fibres with porous walls are ideal for drug loading and release, whereas hollow cores promote target delivery. The natural product clv was loaded into fibres to enhance their antioxidant capabilities. The multifunctional capabilities of the PGFs were evidenced by complementary assessments: antibacterial, cytocompatibility and antioxidant testing *via* DPPH, ABTS, TMB and TPC assays. ROS assays confirmed that PGF-Ga1-clv3 (3 mg mL^−1^) dissolution products mitigate oxidative stress in MC3T3-L1-E1 and HaCaTs.

In contrast to the antioxidant results, clv release did not affect bacterial growth, and only the higher Ga concentrations exhibited antibacterial activity against *E. coli*. No antibacterial activity was observed against *S. aureus*. The accelerated wound closure observed in scratch assays indicates a positive effect of Ga and of clv in wound healing. In HaCaTs, wound closure due to cell migration reached its maximum after 72 h (∼58%). Overall, results indicate that the CTAB-templated PGFs integrate in uniquely porous fibrous structure therapeutic ion release, antioxidant and antibacterial activities, demonstrating their capabilities as multifunctional materials for advanced wound-healing applications. While the results are very promising, further validation through *in vivo* studies is required to assess their actual healing efficacy. Therefore, this work provides a foundational basis for future studies aimed at translating these materials into advanced wound-healing applications.

## Conflicts of interest

There are no conflicts to declare.

## Supplementary Material

MA-007-D6MA00177G-s001

## Data Availability

The data supporting this article are provided in the supplementary information (SI). Supplementary information is available. See DOI: https://doi.org/10.1039/d6ma00177g.
