# Supplementary material for: Effects of gallium and clove oil embedded in porous phosphate coacervate glass fibres on wound healing
Source: Mater Adv. 2026 Jun 11;7(14):7127–45. doi: 10.1039/d6ma00177g (PMC13296919; doi:10.1039/d6ma00177g)
Supplement: MA-007-D6MA00177G-s001 [file MA-007-D6MA00177G-s001.pdf]

This journal is © The Royal Society of Chemistry 2026

Supporting Information

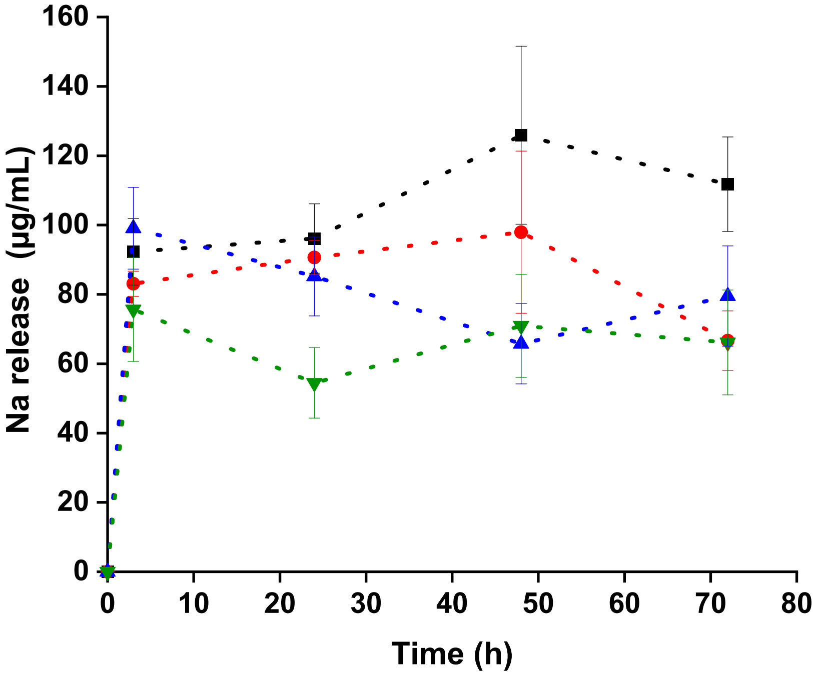

Figure SI_1. Release of Na after PGF-unl and PGFs-GaX immersion in DI water up to 72 h

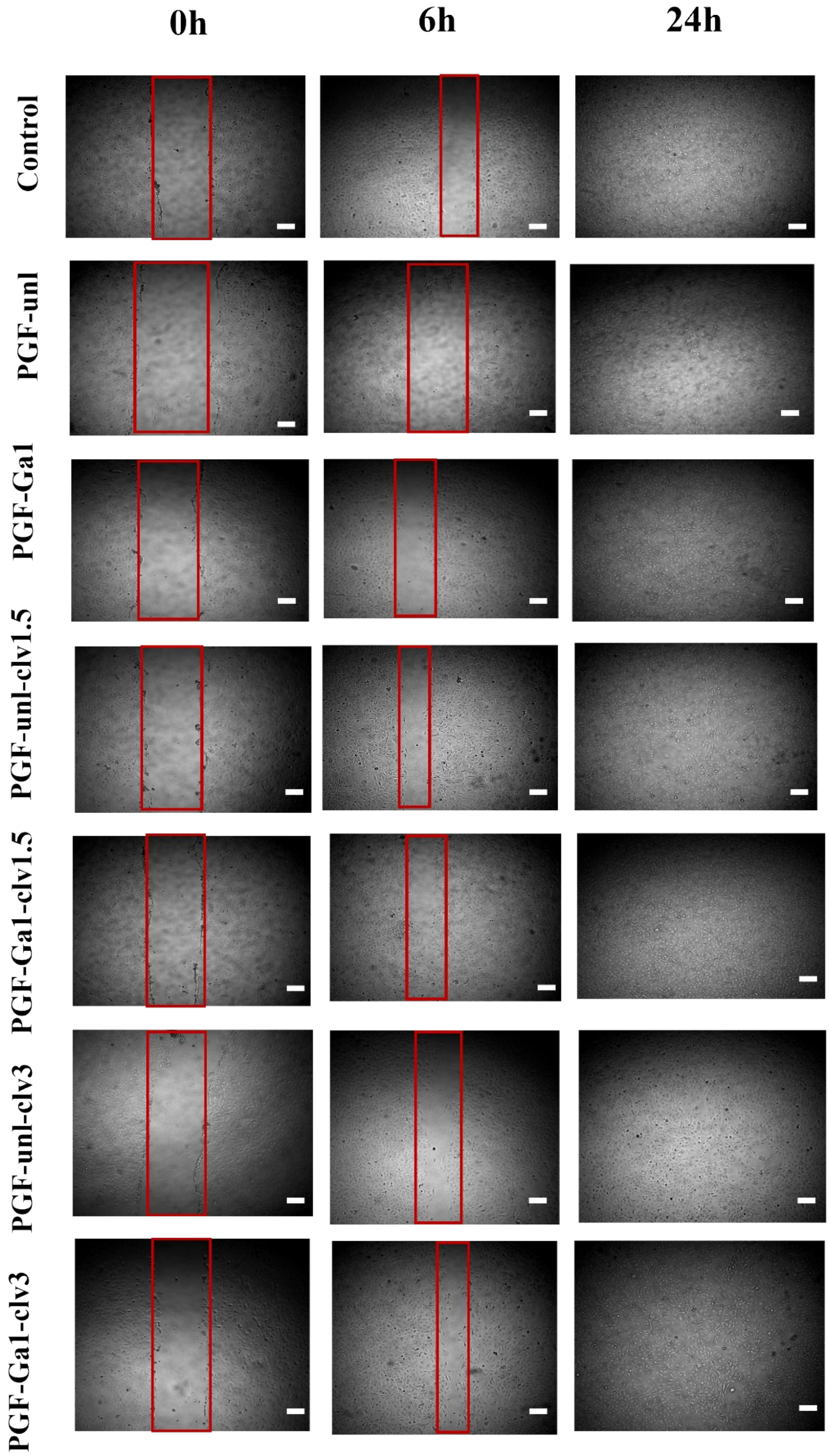

Figure SI_2. In vitro monolayer scratch assay of untreated MC3T3-L1-E1 (control) and those treated with PGF-unl, PGF-Ga1, PGF-unl-clv1.5, PGF-Ga1-clv1.5, PGF-unl-clv3, PGF-Ga1-clv3 after 6 and 24 h. Scale bar: 200 µm

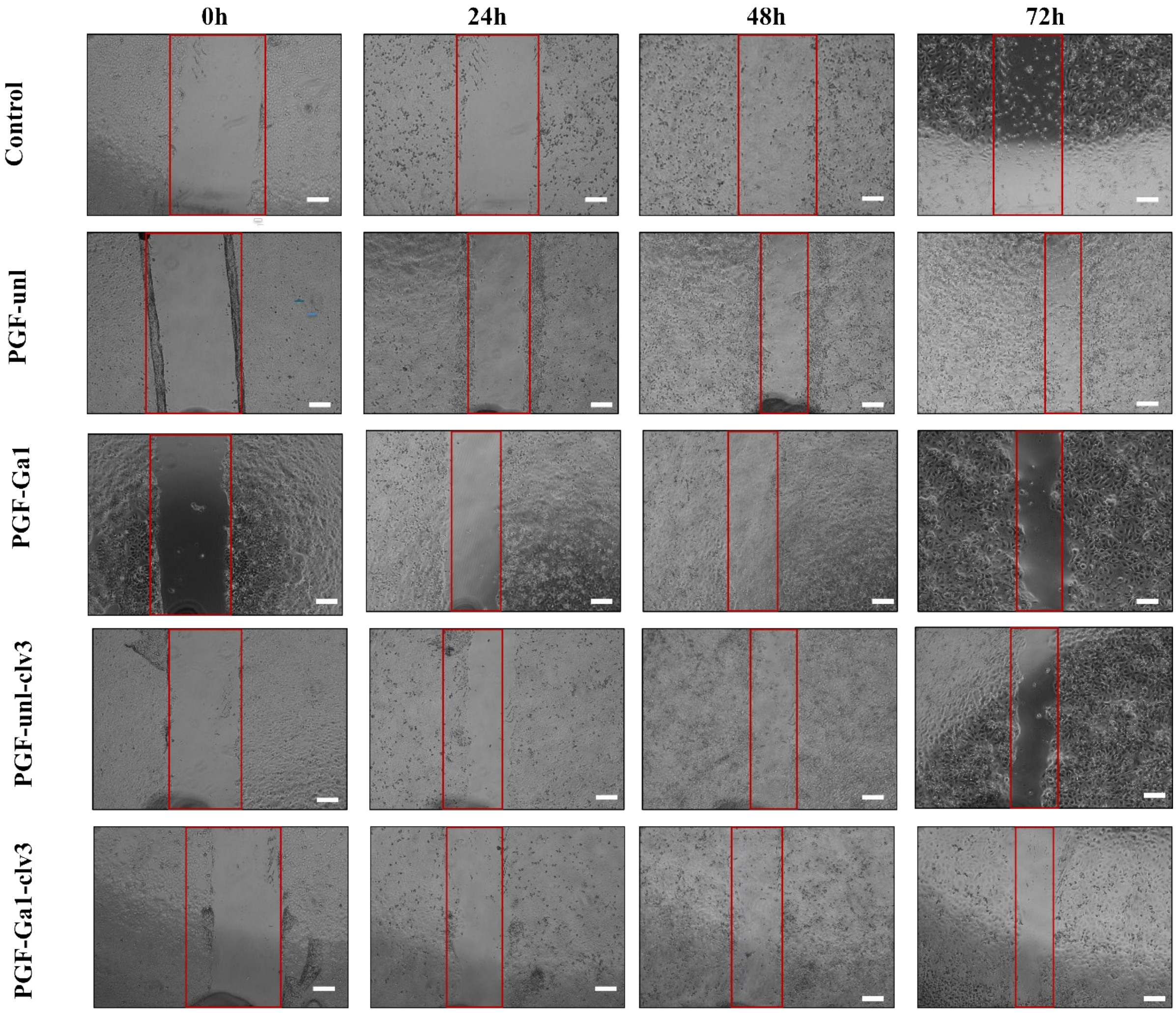

Figure SI_3. In vitro monolayer scratch assay of untreated HaCaTs (control) and those treated with PGF-unl, PGF-Ga1, PGF-unl-clv3 and PGF-Ga1-clv3 after 24, 48 and 72 h. Scale bars: 200 µm

Table SI_1. Scratch width and percentage of wound closure for MC3T3-L1-E1 in contact with PGF dissolution products.

**Sample**	**Scratch width (0 h, µm)**	**Scratch width (6 h, µm)**	**% Closure (6 h)**	**% Closure (24 h)**
Control	650	393	39 %	100 %
PGF-unl	882	702	20 %	100 %
PGF-Ga1	713	450	33 %	100 %
PGF-unl-clv1.5	614	428	30 %	100 %
PGF-Ga1-clv1.5	588	291	50 %	100 %
PGF-unl-clv3	632	324	48 %	100 %
PGF-Ga1-clv3	545	264	51 %	100 %
